# An evolutionary perspective on leaf economics: phylogenetics of leaf mass per area in vascular plants

**DOI:** 10.1002/ece3.1087

**Published:** 2014-07-01

**Authors:** Olivier Flores, Eric Garnier, Ian J Wright, Peter B Reich, Simon Pierce, Sandra Dìaz, Robin J Pakeman, Graciela M Rusch, Maud Bernard-Verdier, Baptiste Testi, Jan P Bakker, Renée M Bekker, Bruno E L Cerabolini, Roberta M Ceriani, Guillaume Cornu, Pablo Cruz, Matthieu Delcamp, Jiri Dolezal, Ove Eriksson, Adeline Fayolle, Helena Freitas, Carly Golodets, Sylvie Gourlet-Fleury, John G Hodgson, Guido Brusa, Michael Kleyer, Dieter Kunzmann, Sandra Lavorel, Vasilios P Papanastasis, Natalia Pérez-Harguindeguy, Fernanda Vendramini, Evan Weiher

**Affiliations:** 1 CNRS, Centre d'Écologie Fonctionnelle et Évolutive (CEFE), UMR 51751919 route de Mende, 34293, Montpellier Cedex 5, France; 2 UMR PVMBT, Université de la Réunion, CIRAD7 chemin de l'IRAT, 94710, Saint–Pierre, France; 3 Department of Biological Sciences, Macquarie UniversityNew South Wales, 2109, Australia; 4 Department of Forest Resources and Institute on the Environment, University of MinnesotaSt Paul, Minnesota; 5 Hawkesbury Institute for the Environment, University of Western SydneyHawkesbury, New South Wales, Australia; 6 Department of Plant Production, University of Milanvia Celoria 2, I-20133, Milan, Italy; 7 Instituto Multidisciplinario de Biología Vegetal (CONICET - UNC) and FCEFyN, Universidad Nacional de CórdobaCasilla de Correo 495, Vélez Sársfield 299, 5000, Córdoba, Argentina; 8 James Hutton InstituteCraigiebuckler, Aberdeen, AB15 8QH, UK; 9 Norwegian Institute for Nature ResearchTungasletta 2, 7485, Trondheim, Norway; 10 Community and Conservation Ecology GroupPO Box 14, 9750, AA Haren, The Netherlands; 11 DBSF, Università degli Studi dell'InsubriaVia J.H. Dunant 3, I- 21100, Varese, Italy; 12 Centro Flora Autoctona, c/o Consorzio Parco Monte Barrovia Bertarelli 11, I-23851, Galbiate (LC), Italy; 13 UR B&SEF CIRAD, TA C-105/D, Campus International de Baillarguet34398, Montpellier Cedex 5, France; 14 INRA UMR 1248 AGIR, Equipe ORPHEEBP 52627 - Auzeville, 31326, Castanet-Tolosan, France; 15 Institute of Botany, Academy of Sciences of the Czech RepublicDukelská 135, CZ-37982, Třeboň, Czech Republic; 16 Department of Botany, Stockholm UniversityStockholm, 106 91, Sweden; 17 Centre for Functional Ecology, University of CoimbraCoimbra, Portugal; 18 Department of Molecular Biology and Ecology of Plants, Faculty of Life Sciences, Tel Aviv UniversityTel Aviv, 69978, Israel; 19 Department of Archaeology, The UniversitySheffield, S1 4ET, UK; 20 Landscape Ecology Group, Carl von Ossietzky University of OldenburgP.O. Box 2503, 26111, Oldenburg, Germany; 21 Landscape Ecology & ConsultingLerchenstrasse 20, 26215, Wiefelstede, Germany; 22 Laboratoire d'Écologie Alpine (CNRS UMR 5553) and Station Alpine Joseph Fourier (UMS-UJF-CNRS 2925), Université Joseph FourierBP 53, F-38042, Grenoble, Cedex 09, France; 23 Laboratory of Rangeland Ecology, Aristotle University of Thessaloniki54124, Thessaloniki, Greece; 24 Department of Biology, University of Wisconsin-Eau ClairePhillips Hall 353, Eau Claire, Wisconsin, 4702-4004

**Keywords:** Brownian model, functional trait, Ornstein–Uhlenbeck model, phenotypic evolution

## Abstract

In plant leaves, resource use follows a trade-off between rapid resource capture and conservative storage. This “worldwide leaf economics spectrum” consists of a suite of intercorrelated leaf traits, among which leaf mass per area, LMA, is one of the most fundamental as it indicates the cost of leaf construction and light-interception borne by plants. We conducted a broad-scale analysis of the evolutionary history of LMA across a large dataset of 5401 vascular plant species. The phylogenetic signal in LMA displayed low but significant conservatism, that is, leaf economics tended to be more similar among close relatives than expected by chance alone. Models of trait evolution indicated that LMA evolved under weak stabilizing selection. Moreover, results suggest that different optimal phenotypes evolved among large clades within which extremes tended to be selected against. Conservatism in LMA was strongly related to growth form, as were selection intensity and phenotypic evolutionary rates: woody plants showed higher conservatism in relation to stronger stabilizing selection and lower evolutionary rates compared to herbaceous taxa. The evolutionary history of LMA thus paints different evolutionary trajectories of vascular plant species across clades, revealing the coordination of leaf trait evolution with growth forms in response to varying selection regimes.

## Introduction

Although leaf morphology has evolved manifold variations across vascular plant species, there is strong evidence of a universal spectrum constraining leaf functioning from rapid resource capture to efficient resource use (Grime et al. 1997; Reich et al. 1997; Díaz et al. 2004; Wright et al. 2004). This worldwide leaf economics spectrum (Wright et al. 2004) consists of the correlated variation among several key plant traits, including leaf mass per area (LMA, the ratio between leaf dry mass and leaf area) which describes the dry mass investment for light interception per unit leaf area (Lambers and Poorter, 1992). LMA captures a central axis of functional variation in plants (Grime et al. 1997; Grime 2001; Westoby et al. 2002; Díaz et al. 2004), correlating negatively with mass-based photosynthetic rate and leaf macronutrient concentrations (Reich et al. 1997; Wright et al. 2004), and positively with leaf life span (Wright et al. 2004). At large spatial scales, LMA varies across climatic gradients, displaying on average higher values in hotter, drier, and higher irradiance habitats, particularly once other factors, such as deciduousness or plant functional type composition, are taken into account (Reich et al. 1997; Wright et al. 2004; Reich et al. 2007; Poorter et al. 2009).

Despite a sound knowledge of the physiological and ecological correlates of LMA, we remain largely ignorant of the evolutionary history that gave rise to the present-day wide variation in this key trait. Two types of overall evolutionary behavior may be expected for a vegetative trait such as LMA: it can either be evolutionary labile and vary independently from the phylogeny across species, which can lead to high functional convergence across clades (Kraft et al. 2007), or it may alternatively display phylogenetic patterns structured by selection within plant lineages (Blomberg et al. 2003; Losos 2008). In the labile trait hypothesis, we nonetheless expect consistent evolution with other plant traits, or syndromes such as growth form. Recent advances have indeed suggested that the evolution of growth form, as a syndrome of multiple traits, has been a major driver of functional trait evolution (Moles et al. 2005; Kerkhoff et al. 2006). Marked taxonomic patterns in LMA would therefore relate more strongly with growth forms than with phylogenetic relatedness. Alternatively, following the second hypothesis, LMA would have evolved under strong selection and be conserved along plant lineages, which would generate a consistent and detectable phylogenetic signal.

Phylogenetic signal can be broadly defined as the information conveyed by the variation in phenotypic trait values within and across clades along a phylogeny. In the present study, we conducted a comprehensive analysis of the evolutionary history of leaf economics across the phylogeny of vascular plants based on the analysis of the phylogenetic signal in LMA. Variation in trait similarity among taxa may reveal patterns of either trait diversification or trait conservatism within clades, that is, the tendency for relatives to be more similar than expected by chance alone. Such macroevolutionary patterns may provide insight into the microevolutionary processes (e.g., selection and drift) shaping trait evolution within lineages (Hansen and Martins, 1996; Hansen 1997; Diniz-Filho 2001).

Variation in leaf mass per area was charted across a dated phylogeny of 5401 species spanning all major clades of vascular plants. Using phylogenetic comparative analyses, we analyzed the phylogenetic signal in LMA and compared observed patterns to expectations based on different models of trait evolution. We show that LMA exhibits low but significant overall phylogenetic conservatism across vascular plant clades. We identify different clades displaying significant phylogenetic patterns of either trait conservatism or diversification. Moreover, we tested the interaction between the evolutionary histories of LMA and growth form across species. We note that potential additional explanatory variables such as biogeography or pedo-climatic conditions were outside the scope of the present study. We found that patterns of LMA evolution consistently differed across growth forms, revealing evidence for a higher conservatism and slower trait evolution in woody species than in herbaceous species.

## Material and Methods

### Trait data

We collected LMA data for vascular plants (*Tracheophyta*) from 180 published and unpublished studies and electronic databases (see Appendix S1). We only retained data collected in the field for outer-canopy leaves measured following standardized protocols (Cornelissen et al. 2003) and for adult plants, to limit ontogenic effects. Mean values were calculated for species with multiple records. The sample set of species and sites represented a wide range of plant communities in most of the climates where vascular plants occur, from Arctic tundra to tropical forest, from hot to cold deserts, and from grassland to woodland. In total, we obtained LMA values for 5401 species in 241 families and 1835 genera: 5239 *Angiospermae*, 81 *Gymnospermae*, 74 *Monilophyta*, and 7 *Lycopodiophyta* (see Fig. 2 for an overview of sample completeness). We checked that the variability in LMA occurred mostly among species compared to within species (across sites): *V*_among_ = 16286 versus *V*_within_ = 1912. In order to limit the influence of extreme values, we performed comparative analyses on log-transformed LMA values that we hereafter note *l*.

### Dated phylogenetic supertree

Phylogenetic relationships between species were described as an informal supertree based on published phylogenies (Bininda-Emonds 2004). We used the tree of Chaw et al. (2000) as a backbone for relationships between major vascular plant clades and between families within *Gymnospermae* on which we branched family-level trees for the *Angiospermae* (Davies et al. 2004), *Monilophyta* and *Lycopodiophyta* (Wikström and Kenrick 2001; Smith et al. 2006). We delimited major clades *Tracheophyta* according to the phylogenetic nomenclature of Cantino et al. (2007). Family names in *Angiospermae* were matched to the latest phylogeny by the Angiosperm Phylogeny Group (APG III 2009). Within 34 large *Angiospermae* families, we resolved relationships among genera using published family phylogenies (see Appendix S1). Species were branched as polytomies within genera. The resulting supertree had 1675 internal nodes among which 58% were resolved as bifurcations (nodes with two daughter clades). Below the genus level, the percentage of bifurcations among ancestral nodes rose to 75%. In order to estimate branch lengths, we dated the ancestral nodes of the supertree in a two-step procedure. We first dated well-identified nodes in the supertree using published ages. A total of 187 ancestral nodes matched dated nodes from the literature (11% of internal nodes). For clades in *Angiospermae*, ages were taken from a comprehensive update of divergence times based on the analysis of sequence data (Bell et al. 2010). We completed these ages with estimates derived from several sources for non-angiosperm clades (Wikström et al. 2001; Bremer et al. 2004; Janssen and Bremer, 2004; Anderson et al. 2005). Node ages ranged from 4.4 Myr for the Juglandaceae family to 535 Myr for the tree root, namely the *Tracheophyta* divergence (mean: 88.1 Myr SD: 75.3 Myr). Second, node ages were estimated using the *bladj* module of the Phylocom software (Webb et al. 2007). This procedure sets branch lengths by placing the nodes evenly between dated nodes, and between dated nodes and tips (of age 0). This has the effect of minimizing variance in branch length, within the constraints of dated nodes (Webb et al. 2007). The phylogenetic distance between two species was estimated by the age of their most recent common ancestor.

An alternative method would have been to reconstruct a phylogeny from molecular data. For large datasets, however, it is difficult if not impossible to obtain resolved phylogenies at species level. A recent study compared phylogenetic patterns in phenology for *ca*. 4000 species (Davies et al. 2013) using phylogenetic analyses of both an informal supertree, such as the one constructed here, and a phylogenetic tree obtained from molecular data and resolved at genus level. The patterns described were similar and led to the same conclusions. We also explored this option, but were only able to locate genetic data for about 40% of species. We eventually chose to retain a much wider coverage but lower resolution.

### Models of continuous trait evolution

We tested and compared alternative evolutionary hypotheses by fitting different evolutionary models to our data. Models of trait evolution describe the macroevolutionary patterns which would be expected from hypothetical microevolutionary processes (Hansen and Martins, 1996). First, we considered a simple model of Brownian motion evolution (BM), which assumes that the trait evolves independently in each lineage by means of genetic drift and/or directional selection under environmental conditions fluctuating randomly and rapidly compared to evolutionary time (Hansen and Martins, 1996; Diniz-Filho 2001). The BM model supposes evolution by random motion of trait values at a constant rate *σ* along the branches of the phylogenetic tree and thus mimics the effects of drift (Hansen and Martins, 1996; Freckleton et al. 2002): during an infinitesimal period *dt*, the variation in the trait value *l* is *dl* = *σ*^2^*dt*, where parameter *σ* controls the magnitude of stochastic perturbations during the course of evolution, or drift (Hansen 1997).

Second, we considered models of LMA evolution incorporating both stabilizing selection and drift (Hansen 1997). These models conform to evolution following an Orstein-Ulhenbeck (OU) process: during an infinitesimal period *dt*, the variation of the trait value sums as the effects of drift, *σ*^2^*dt* as in BM, and selection toward a phenotypic optimum *θ*: *dl* = −*α*(*l*−*θ*) + *σ*^2^*dt*, where the parameter *α* controls the rate of adaptive evolution to the optimum (Hansen 1997). Parameter *α* measures the magnitude of a supposed selective force. Note that a null value of *α* leads to the BM model, which thus appears as a special case of OU model. Interestingly, OU models allow for different selective optima to be specified within different clades along a single phylogenetic tree (Hansen 1997; Butler and King, 2004): changes in the selective optimum mimic variation in the selection regime along the phylogeny. After a change in the selection regime, evolution unfolds independently within each of the lineages. We calibrated three OU models simulating evolution with one (OU1), three (OU3), or five (OU5) phenotypic optima (Butler and King, 2004) (see Fig. 5). Models were fitted over the complete set of species, as well as on subsets of species including only herbaceous (forbs and graminoids) or woody (shrubs and trees) growth forms (see SOM for details on growth forms and model fitting).

Finally, we also considered a null model of phylogenetically independent evolution (PI) to test for the existence of phylogenetic structure in the trait data. The PI model ignores phylogenetic relatedness across species as if they were placed at equal distance on a star phylogeny. It serves as a null hypothesis which supposes the absence of phylogenetic covariance in the trait distribution.

### Treewise phylogenetic signal

We calculated three statistics to quantify the magnitude and test the significance of the overall phylogenetic signal in the phylogeny of vascular plants (see SOM for details): Pagel's *λ* (Pagel 1999), Blomberg's *K*-statistic (Blomberg et al. 2003), and phylogenetic autocorrelation measured by Moran's index (Gittleman and Kot, 1990). These statistics provide information on the likelihood of the phylogenetic pattern with respect to models of trait evolution. In Pagel's approach, a value of *λ* = 0 indicates evolution independent of phylogeny (PI), while a value of *λ* = 1 indicates that the phylogenetic pattern conforms to Brownian motion on the given phylogeny (Freckleton et al. 2002). The *K*-statistic measures the degree to which a phylogenetic tree correctly describes the covariance structure observed in the data compared to a BM model along the candidate tree: *K* > 1 indicates higher conservatism than in the BM case. Finally, Moran's index, *I*, quantifies the similarity in LMA among species with respect to their phylogenetic distance (Gittleman and Kot, 1990; Diniz-Filho 2001). It is a measure of the covariation of trait values across species weighted by the phylogenetic distance between them: *I* varies from 1, indicating strong positive association (similarity across close relatives) to −1, indicating strong negative association. It has an expected value of 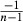, where *n* is the number of tips, under the null hypothesis of no correlation between trait values and phylogenetic distance. We also calculated *I* within different classes of divergence time (i.e., age of the most recent common ancestor) which were chosen to ensure a sufficient number of observations within each class. This was used to build a resulting phylogenetic correlogram, which represents how trait similarity among species varies with the time since their divergence. In theory, this temporal pattern allows one to discriminate the BM from the OU models (Hansen and Martins, 1996; Diniz-Filho 2001): a linear decrease in similarity with time conforms to a model of evolution by Brownian motion, whereas an exponential decrease indicates evolution by drift and stabilizing selection as in the OU models (Hansen and Martins, 1996). Thus, we adjusted linear and exponential fits to the observed empirical patterns using nonlinear least squares and compared the fits using a likelihood ratio test. Similar analyses were also performed within subsets corresponding to the different growth forms.

### Phylogenetic signal at clade level

We analyzed the phylogenetic signal at clade level using the analysis of traits procedure (AOT Ackerly, and Kembel, 2007). This analysis can detect functionally diversifying and conservative ancestral splits in trait values across daughter clades (Ackerly and Nyffeler, 2004; Moles et al. 2005). At each divergence in the phylogeny (i.e., node of the tree), ancestral mean trait values are calculated according to the procedure described in Felsenstein (1985). We then calculated the divergence width statistic, *DW*, which measures the degree of divergence (or trait radiation) between the child clades of each node. An ancestral node was considered as diversifying when the corresponding *DW* value was higher than expected at random, and as conservative when *DW* was lower than expected at random. Significant divergence widths were detected by testing the statistic against the null hypothesis PI using permutations (*n* = 10^5^) and correcting for multiple comparisons (Benjamini and Hochberg, 1995). Significantly diversifying nodes, that is, with higher *DW* than expected under the null hypothesis (PI), indicate past diversifying events (trait radiation) followed by subsequent conservatism within daughter clades. On the other hand, significantly conservative nodes correspond to divergences of low amplitude between daughter clades thus displaying similar trait values and phylogenetic conservatism thereafter.

## Results

**Figure 1:**
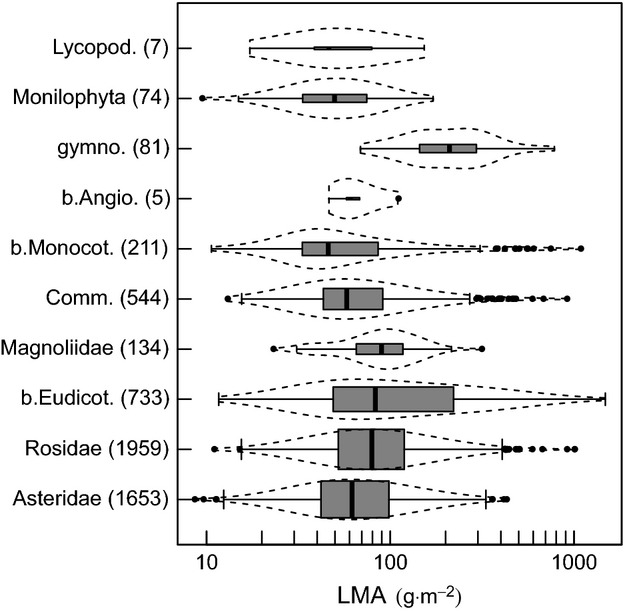
Leaf mass per area (LMA) within clades. Distribution of LMA (g.m^−2^) with respect to the major monophyletic clades of vacular plants (*Tracheophyta*) in increasing order of divergence time (letter “b” refers to basal clades, numbers in brackets indicate the number of species in the clades, *n*). Boxes and vertical lines indicate the interquartile range and the median in each category. Box height is proportional to 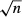 (see Table S1). Dotted lines represent the smoothed distribution within each category, and crosses indicate outliers.

**Figure 2:**
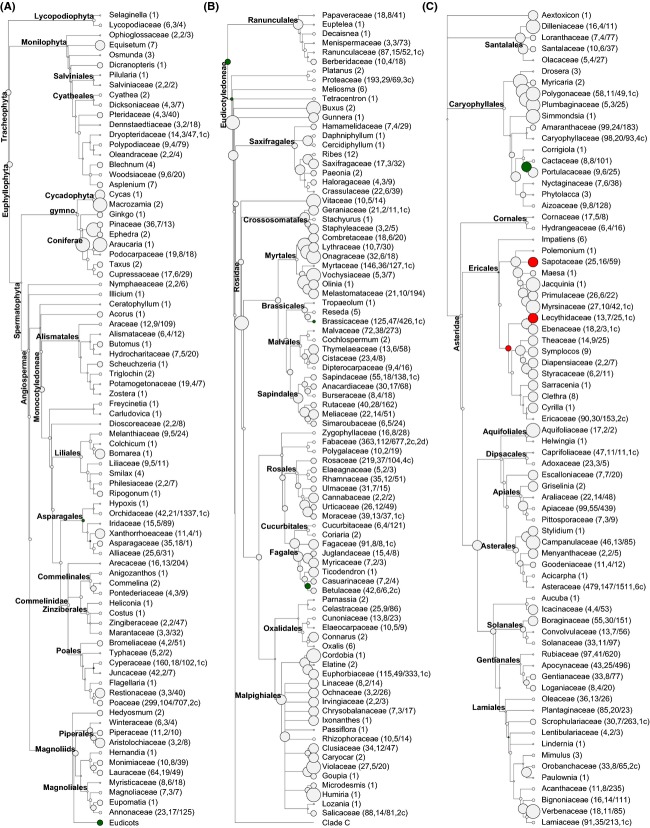
Phylogenetic tree of the sampled extant species. The tips of the tree correspond to botanical families, except for families with one genus only, in which case the genus is indicated. Symbol size represents the clade mean LMA in five classes of increasing values: < 50, [50−75], [75−100], [100−150], and ≥ 150 g.m^−2^. Red symbols indicate significant conservative evolutionary splits (with divergence width, *DW*, significantly lower than expected at random), green symbols represent significant diversifying divergences (*DW*, significantly higher than expected at random). Numbers in parentheses are: number of sampled species, number of sampled genera / number of genera in the family, according to Kew's classification. Where appropriate, the number of significant divergences within families is indicated in parentheses as “c" for conservative and “d" for diversifying. Tracheo.: vascular plants (Tracheophytes), Euphyllo.: megaphyll plants, Spermato.: seed plants (Spermatophytes). Branch lengths are indicative.

Across the complete dataset, the mean LMA value was found to be 75.6 g.m^−2^, with values ranging from 8.8 g.m^−2^ in *Impatiens parviflora* (*Balsaminaceae*) to 1479.1 g.m^−2^ in *Hakea leucoptera* (*Proteaceae*). LMA ranges largely overlapped across the major clades in *Tracheophyta*, but on average, *Lycopodiophyta* and *Monilophyta* (ferns) exhibited the lowest LMA, while *Gymnospermae* showed the highest LMA compared to the other major clades (Fig. 1; Table S1). Within the *Angiospermae*, *Monocotyledoneae* tended to have lower LMA values than *Eudicotyledoneae* (Fig. 1). Finally, within *Eudicotyledoneae* (80% of sampled species; Table S1), basal clades showed higher LMA values than more recently diverged clades (*Asteridae* and *Rosidae*; Figs 1 and 2).

**Figure 3:**
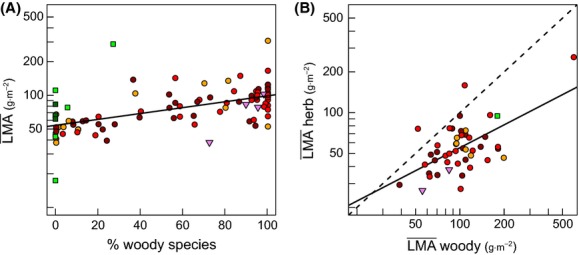
Phylogenetic and growth form signal in LMA across and within angiosperms families: (A) mean LMA, *LMA*, versus proportion of woody species (shrubs and trees; best linear fit: *r*^2^ = 0.24, *P* < 10^−4^); (B) Mean LMA of herbaceous species (forbs and graminoids) versus mean LMA of woody species within families (dashes: 1 to 1 line; best linear fit: *r*^2^ = 0.26, *P* < 10^−3^). Symbols and colors indicate major clades of angiosperms: ○ *Eudicotyledoneae* (orange: basal families, red: *Asteridae*, dark red: *Rosidae*); □ *Monocotyledoneae* (green: basal families; dark green: *Commelinidae*); ▽ *Magnoliidae*. Only families with over 10 sampled species were considered.

At the family level, within angiosperms, the proportion of woody species in a family explained 24% of the variation in family mean LMA values (Fig. 3A). This was due to the tendency for woody species to have higher LMA than herbaceous species (Fig. S1). Nevertheless, the mean LMA of herbaceous and woody species belonging to the same family was found to be positively related (Fig. 3B). This is a first indication of the existence of phylogenetic conservatism in LMA values within families which seems to interact with the overall effect of growth forms on LMA.

Overall, common statistics characterizing the phylogenetic signal in LMA indicated low but significant similarity between close relatives, hence low conservatism, as well as little departure from the expected pattern of evolution following BM: Blomberg's *K*–statistics (Blomberg et al. 2003) was 0.093 (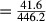), Pagel's *λ* (Pagel 1999) was estimated to 0.946, and Moran's phylogenetic index (Gittleman and Kot, 1990) was 0.034 (see Material and Methods for interpretations).

In order to identify specific clades showing significant evidence of conservatism or diversification, we estimated the divergence width, *DW*, or magnitude of divergence in LMA values across daughter clades at each evolutionary node (Ackerly and Nyffeler, 2004; Moles et al. 2005). When the 1675 internal nodes of the phylogeny were considered, *DW* significantly differed from random in just 62 cases (3.7%). Of these 62 nodes, 12 corresponded to diversifying nodes with trait radiation (Table 1, Fig. 2), and 50 to conservative nodes with trait clustering across daughter clades. Conservative nodes mostly occurred among terminal nodes (genera, Table S4), indicating conservatism in LMA values among congeneric species, and at the basis of four larger clades including the *Lecythidaceae* and *Sapotaceae* families (Table 1). Cladogenesis at the base of these four clades led to sister clades more homogeneous than expected at random with regard to LMA. Compared to splits showing conservatism in LMA, diversifying nodes were typically older (70.2 Myr old vs. 17.2 Myr old on average), and thus account for much of the evolution of the basic range of leaf functioning. The most extreme diversifying events in LMA were ancient divergences followed by subsequent conservatism within descending clades (Table 1, Fig. 2). Diversification in LMA thus seems to have occurred early during the course of vascular plant evolution. We investigated whether this pattern was driven by the contrast between the large majority of angiosperms in the dataset and the early-diverging lycophyte, fern, and gymnosperm clades by running the analysis on the dataset restricted to angiosperms. When early-diverging clades were excluded, the same early evolutionary splits were identified as diversifying within the angiosperm clade (Table 1).

**Table 1:** Significant evolutionary splits below genus level (see Table S4 for results concerning genera). Clades are named after taxonomic names, unless undefined. Sister clades, separated by “/", descend from the focal nodes (not detailed for the polytomies at the base of the Lecythidaceae and Sapotaceae families); *Xantho*. indicates the *Xanthorreaceae* family. Age: estimated age of the evolutionary split in Myr. ♯ tips: number of descending tips (size of the clade). LMA: respective mean LMA value within sister clades, in g.m^−2^, except for Lecythidaceae and Sapotaceae (mean LMA within parent family). Woody (%): respective proportion of woody species (shrubs/trees) within both sister clades. DW: divergence width of the split. Diversifying and conservative splits showed significantly higher and lower DW compared to random, respectively

Name	Sister clades	Age (Myr)	♯ tips	LMA (g.m^−2^)	Woody %	DW
*Diversifying splits*						
*Eudicotyledoneae*	*Ranunculales* / rest of *Eudicotyledoneae*	144	4345	51/78	11/55	0.598
–	*Proteales* / sister clade	142	4225	301/73	100/53	0.619
*Asparagales*	*Alliaceae* + *Asparagaceae* + *Xantho*. / *Orchidaceae* + *Hypoxidaceae*	108	129	94/43	6/0	0.682
*Ericales*	*Impatiens* / rest of *Ericales*	91	249	17/96	0/81	1.316
–	*Alliaceae* + *Asparagaceae* / *Xantho*.	74	71	72/287	3/27	0.931
–	*Cyperaceae* / *Juncaceae*	61	202	66/83	0/0	0.528
–	*Scrophulariaceae* / 9 families of *Lamiales*	55	208	101/56	53/29	0.662
–	*Mirbelieae* tribe / sister clade in *Fabaceae*	53	192	145/52	100/17	0.639
*Brassicaceae*	*Capparoideae* subfamily / rest of *Brassicaceae*	43	125	161/44	100/2	1.081
–	*Betulaceae* / *Casuarinaceae*	30	49	65/445	100/100	1.442
–	*Portulacaceae* / *Cactaceae*	22	17	66/523	0/13	1.467
–	*Acacia* / *Ingeae* tribe in *Fabaceae*	20	63	195/83	100/100	0.659
*Conservative splits*						
–	*Ebenaceae* + *Lecythidaceae* / sister clade	69	162	102/113	97/96	<10^−3^
*Sapotaceae*	16 sister clades	45	25	124		0.195
*Lecythidaceae*	7 sister clades	35	13	99	100	0.099
	*Sagina* / *Bufonia tenuifolia* in *Caryo*.	16	7	63/63	0/0	<10^−3^

**Figure 4:**
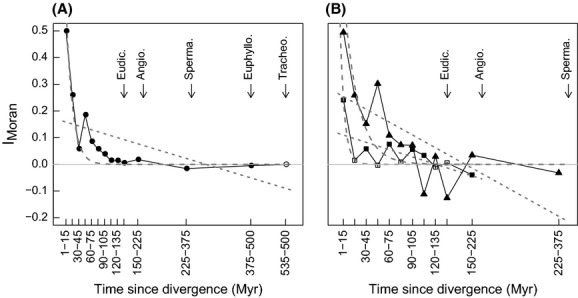
Similarity in LMA as a function of divergence time. Moran's index (*I*_*M*_) in classes of divergence time (Myr) for (A) the complete species set (*n* = 5401), and (B) the herbaceous (□, *n* = 2417) and woody (Δ, *n* = 2564) growth forms. Filled (resp. open) symbols indicate (non-)significant correlations (*α*level: 0.05). Error bars were smaller than symbol size and therefore not represented. Dotted lines represent linear fits (Brownian Motion model, BM); dashed lines represent exponential fits (Ornstein–Uhlenbeck model, OU). Arrows indicate major evolutionary nodes; Tracheo.: vascular plants, Euphyllo.: megaphyll plants (ferns + seed plants), Sperma.: seed plants, gymno.: *Gymnospermae*, Angio.: *Angiospermae*, Magno.: *Magnoliidae*.

Phylogenetic correlograms further revealed how similarity in LMA among species rapidly decreases with divergence time (Fig. 4). On average, the older the most common ancestor, the lower the correlation in LMA tip values. Similar values for recent divergences (low divergence time) showed that, on average, any two close relatives among woody species were closer functionally than any two close relatives among herbaceous species. More generally, woody species showed a greater overall similarity through time and hence a higher degree of conservatism in LMA than herbaceous species (Fig. 4). *Angiospermae*, representing 97% of sampled woody species (Table S1), were mostly responsible for this pattern. Moreover, functional similarity in LMA decreased differently in woody species (shrubs and trees) and in herbaceous species (forbs and grasses; Fig. 4B), suggesting that resource-use strategies have evolved conjointly with growth form in vascular plants.

**Figure 5:**
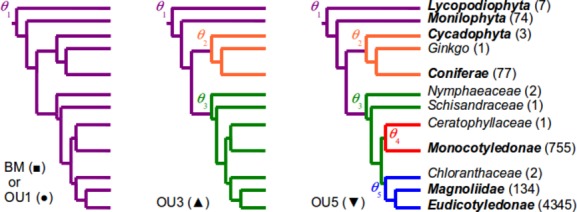
Alternative models of LMA evolution based on Brownian motion (BM, ▪) and Ornstein–Uhlenbeck process with one (OU1, •), three (OU3, ▴), or five (OU5, ▾) phenotypic optima indicated by different colors. Only major and basal clades of *Tracheophyta* are represented (arbitrary branch lengths; numbers indicate clade size). Models were fitted on the complete phylogeny and on the herbaceous and woody species groups separately by pruning the phylogeny accordingly (see Fig. S6).

We further analyzed the evolution of LMA by means of models of continuous trait evolution. The models supported the hypothesis of stabilizing selection around some optimal trait values against evolution of LMA by drift only: the OU performed better than the simple BM models according to BIC ranking (Table S3). This conclusion, drawn for the complete phylogeny, was also supported for woody and herbaceous growth forms considered separately. The better fit of OU models was consistent with the linear trends of decreasing similarity with divergence time observed in the empirical correlograms (Fig. 4), as opposed to an expected exponential decrease in the case of BM models (Diniz-Filho 2001). Second, models incorporating different selection regimes (Fig. 5), that is, stabilizing selection around different optimal trait values (Butler and King, 2004) performed better than single-optimum models (Table S3), supporting the existence of shifts in LMA selection regimes between *Angiospermae* versus *Gymnospermae*, and between *Monocotyledonae* versus *Eudicotyledonae*. Finally, such shifts in selective regimes were also observed within each growth form analyzed separately: between the *Angiospermae* and *Gymnospermae* clades for woody species, and between the *Monocotyledonae* and *Eudicotyledonae* clades for herbaceous species (Fig. 5, Table S3). The best identified models explained, respectively, 26.3%, 25.9%, and 42.3% of the null deviance for the complete, woody, and herbaceous datasets.

**Figure 6:**
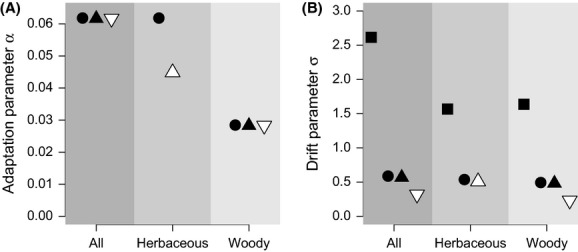
Parameter estimates for models of trait evolution: (A) parameter *α* estimates the rate of adaptation (Hansen, 1997), (B) parameter *σ* estimates the magnitude of perturbations not due to selection (Hansen, 1997). Symbols indicate different models: Brownian motion (▪) and Ornstein–Uhlenbeck models, with one (•), three (▴), or five (▾) selective optima (see Fig. 5 for model description). White symbols indicate the best models according to BIC ranking (Table S3). Error bars were too small to be represented.

Orstein-Ulhenbeck models allow us to estimate the rate of adaptation to phenotypic optima in LMA (*α* parameter on Fig. 6A, Hansen 1997). The quantity 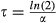 measures the time taken by a phenotype evolving under a new regime to move halfway from its ancestral state (Hansen 1997). We found that this time was 15.5 Myr for herbs and 24.5 Myr for woody species, that is, a longer time scale along woody lineages for new selective conditions to be more influential than constraints from the ancestral state.

## Discussion

The evolutionary history of a key plant functional trait, leaf mass per area (LMA), was investigated across a large set of vascular plant species. Using comparative phylogenetic methods and a global trait database of over 5000 species, we detected that leaf strategies were overall weakly, albeit significantly, conserved during the diversification of vascular plants. This low conservatism appeared to be associated with multiple regimes of stabilizing selection, that is differing adaptive optima across the major clades. Importantly, we highlight the strong interaction between the evolution of growth forms and of leaf strategies, with woody species displaying slower leaf diversification and higher conservatism in LMA than herbaceous plant species.

Leaf mass per area appeared significantly but weakly conserved. This low phylogenetic signal captures the fact that distantly related species may occupy similar positions along the leaf economics spectrum. The large variability found within clades here evidenced a pattern of functional convergence across clades over a broad phylogenetic scale. However, significant levels of trait conservatism imply that, to a certain extent, closely related species do tend to invest dry matter similarly within leaf tissues and hence occupy nearby positions along the leaf economics spectrum. In particular, evolutionary splits identified as conservative nodes were mostly found at the level of genera. As might be expected, the degree of similarity in resource-use strategies varied with the length of the period of common evolution along lineages (Hansen and Martins, 1996). This pattern indicates a temporal dimension to the diversification of resource-use strategies among species. When considering large time scales, the phylogenetic pattern in LMA displayed early diversification associated with the divergences between extant ferns and lycophytes on the one hand and seed plants (*Spermatophytae*) on the other, and between *Angiospermae* and *Gymnospermae* (Fig. 4). Within the *Angiospermae* clade, diversity in LMA reflects the wide adaptive radiation of flowering plants into a range of ecological strategies, involving diverse growth forms and ecological niches both within and across habitats (Ricklefs and Renner, 1994; Losos 2008). By contrast, the consistency of leaf economics in the other major clades may reflect both stabilizing selection (around a given optimum) as well as their marginalization to particular ecological situations during the adaptive radiation of the *Angiospermae*. In fossil floras, basal plant clades display a higher diversity of growth forms than is currently observed among extant species: treelike plants existed within *Lycopodiophyta*, as well as ruderal herbaceous taxa within gymnosperms (Rothwell et al. 2000). The fossil record thus reveals that extinction events also shaped the spectrum of resource-use strategies that exist today in vascular plants.

Models of continuous trait evolution supported the hypothesis that stabilizing selection shaped the phylogenetic pattern in LMA, against the hypothesis of evolution by drift alone. Given the breadth of sampling in our dataset, over a large taxonomic scale and a range of ecological situations, the stabilizing selection we detected likely reflects selection against extreme phenotypes that infringe structural and physiological limitations to LMA. The phylogenetic patterns evidenced here showed simultaneously conservatism, mostly within genera, and convergence across large clades. Congruence between radiation and restrictive environmental conditions, such as within the Proteales, also suggests strong filtering effects that may lead to conservatism at larger phylogenetic scales and functional distinctiveness (Cornwell et al. 2014). Physical conditions *in natura* set constraints on plant strategies that may directly or indirectly affect achievable LMA values. A recent study of the evolution of the leaf economics spectrum highlighted the nature of evolutionary forces on LMA (Donovan et al. 2011). The authors argued that selection in general, rather than genetic constraints, directed the evolution of LMA. As an example, direct selection could influence LMA via biomechanical limits on the amount of dry matter needed to support and maintain a planar photosynthetic surface. Indirect selection could result from selection against unfit LMA values in relation to major trade-offs in plant strategies (Donovan et al. 2011). Furthermore, the models of trait evolution determined different optimal phenotypes across major clades of vascular plants. These findings indicate that these clades have evolved under distinct selective regimes (Butler and King, 2004) that may be interpreted as ecophysiological constraints that support different LMA optima. These constraints can be understood as trade-offs in plant traits that may render particular values of LMA unfit (Donovan et al. 2011). Changes in functional optima along the phylogeny have likely resulted from a combination of changing selection pressure effects, trade-offs among axes of plant variation imposed by these pressures, and historical contingency or ancestry. These variations in LMA optima across clades also existed within growth forms. Hence, major divergences during plant evolution gave birth to clades that in time evolved leaf strategies optimized in response to selective regimes that have imprinted the evolution of both woody and herbaceous lineages within those clades.

Recent analyses of molecular sequences have shown that woody species have evolved more slowly than herbaceous species (Smith and Donoghue, 2008) and taller species more slowly than shorter species (Lanfear et al. 2013), presumably because of their longer generation times. However, the issue of whether molecular evolutionary rates coincide with phenotypic evolutionary rates is somewhat controversial (Bromham et al. 2002). Here, we found evidence supporting different evolutionary rates for LMA across growth forms, with higher rates in herbaceous compared to woody species. The woody syndrome occurred with higher and more consistent functional similarity among lineages that shared the habit. We suspect that allometry in carbon allocation patterns inherent to woody plants has constrained the variability of achievable resource-use strategies and led to higher conservatism along woody lineages through time. Woody plants must allocate carbon extensively to secondary cell wall thickening (including lignification), particularly in mechanical tissues and secondary xylem tissue. This requisite feature of carbon allocation constrains the resource-use strategies of woody plants. Vascular plants in general exhibit a three-way trade-off between: small leaves of high LMA, large leaves of intermediate LMA, and small leaves of low LMA (Pierce et al. 2013). This latter combination, common among ruderal herbaceous species and aquatic plants, is rarely exhibited by woody species. Furthermore, many of the extremely high LMA gymnosperm taxa exhibit phylogenetic constraint based on the structure and functioning of the xylem: tracheary elements of most extant gymnosperm families consist of solely tracheids, and not of tracheae that would provide sufficient internal translocation to support fast growth rates and extensive evapotranspiration from broad leaves. Gymnosperms therefore typically exhibit a suite of sclerophytic traits, such as a thick leaf endodermis, alongside resin canals to guard against predator and pathogen attack, embodied in “needle" leaves of high LMA. These adaptations require an investment in carbon and mineral resources in leaf mass that cannot then be allocated to low LMA leaves typically associated with fast growth rates in ruderal angiosperms. Thus, the investment of carbon in wood and additionally the type of wood produced both provide constraints to woody plant resource-use strategies.

The observed patterns in the evolution of LMA raise the issue of selective pressures resulting in different selection regimes at large evolutionary scales. Fossil evidence suggests that large spatiotemporal scale changes in climate and disturbance regime were partially responsible for the diversification of vascular plants (Stebbins 1974; Jacobs et al. 1999) and this was likely paralleled by diversification in LMA. Recent evidence showed that plant species tend to be more woody and taller toward the tropics (Moles et al. 2009). The herbaceous syndrome might have evolved under more temperate climatic conditions (Ricklefs and Renner, 1994), which suggests that the woody/herbaceous dichotomy represents a major geographical and macroevolutionary dichotomy between climate extremes (Zanne et al. 2013). Evolutionary trends in LMA probably arose in various ways, as this trait integrates several different aspects of leaf morpho-anatomy (Garnier and Laurent, 1994). The interaction of LMA evolutionary patterns with growth forms also suggests strong linkages between leaf and stem traits in the evolution of resource-use strategies (Zanne et al. 2013).

By exploring the evolutionary history of LMA we have revealed different evolutionary trajectories involving coordination and trade-offs between leaf traits and plant traits related to growth form. As a consequence, different optimal phenotypes of resource-use strategies have evolved in vascular plants setting fundamental limits on the structure and physiology of organisms and therefore on ecological processes within communities. Continuing advances in genomics (Leebens-Mack et al. 2006), phylogenetic informatics (Smith et al. 2011), and global trait databases (Kattge and the TRY consortium, 2011) promise to bring further insight into the evolutionary patterns of a range of plant functional traits (e.g., plant height, wood density, roots traits). Confronting these multiple evolutionary patterns will provide a powerful general narrative concerning the mode and speed of evolution of trait variation and distinct plant ecological strategies.

## Supporting Information

**Appendix S1.** References.

**Figure S1.** Leaf Mass per Area (LMA) within growth forms. Distribution of LMA (g.m^−2^) with respect to the different growth forms in order of increasing mean LMA values. In brackets: *n*, number of species per growth form (Table S1). **Figure S2.** Detailed distribution of Leaf Mass per Area (LMA) within ferns and lycophytes. **Figure S3.** Leaf Mass per Area (LMA) in the subsample of (a) herbaceous species for which life history was unambiguous (annual: mean: 45g.m ^−2^/ coefficient of variation: /0.51, *n* = 545; and perennial: 65/0.82, *n* = 921) and of (b) woody species for which leaf phenology was unambiguous (deciduous: 79/0.38, *n* = 522; evergreen: 174/0.76, *n* = 969). **Figure S4.** LMA at the level of families: (a) Mean LMA (LMA in g.m^−2^) across extant species within families vs estimated age of families (in Myr); (b) Coefficient of variation of LMA within families vs estimated age. **Figure S5.** Correlogram of LMA showing Moran's I (IM) within taxonomic levels, for the complete sample (black line and symbols, *n* = 5401 species), and for the woody (dark gray, *n* = 2564 species) and herbaceous (light gray, *n* = 2417 species) growth forms. **Figure S6.** Alternative models for LMA evolution in woody (top) and herbaceous species (bottom).

**Table S1.** Growth forms and clades. **Table S2.** Results from family-level linear models of the mean and coefficient of variation (CV) of log-transformed mean LMA values, l, within *Eudicotyledonae* families. **Table S3.** Summary of evolution models for LMA. **Table S4.** Genera associated with significant conservative divergences.
