# Supplementary material for: An evolutionary perspective on leaf economics: phylogenetics of leaf mass per area in vascular plants
Source: Ecol Evol. 2014 Jul 1;4(14):2799–811. doi: 10.1002/ece3.1087 (PMC4130440; doi:10.1002/ece3.1087)
Supplement: Supplementary file 1 — Appendix S1. References. [file ece30004-2799-SD1.pdf]

References:

Data [supplementary references to Glopnet[1] and LEDA[2] databases]:

Abrams MD & Kubiske ME (1990) *For Ecol Man* 31:245–253; Abrams MD & Mostoller SA (1995) *Tree Phys* 15:361–370; Austin AT & Vitousek PM (1998) *Oecol* 113:519–529; Bigelow SW (1993) *J Veg Sci* 3:401–408; Bonal D *et al* (2000) *Oecol* 124:454–468; Caccianiga M *et al* (2006) *Oikos* 112:10–20; Campanella MV & Bertiller MB (2008) *J Veg Sci* 19:75–85; Castro-Diez P *et al* (1997) *Tr - Struc Func* 11:127–134; Choler P (2005) *Arc Ant Alp Res* 37:444–453; Choong MF *et al* (1992) *New Phyt* 121:597–610; Cunningham SA *et al* (1999) *Ecol Monog* 69:569–588; Davies S (1998) *Ecol* 79:2292–2308; Díaz S *et al* (2004) *J Veg Sci* 15:295–304; Falster DS & Westoby M (2005) *J Ecol* 18:337–343; Falster DS & Westoby M (2005) *Oikos* 111:57–66; Field C (1983) *Oecol* 56:341–347; Fonseca CR *et al* (2000) *J Ecol* 88:964–977; Garnier E *et al* (2006) *Ann Bot* 99:967–985; Givnish TJ *et al* (2004) *Am J Bot* 91:228–246; Gohel N (2001) Master's thesis Université de Montpellier II Sciences et Techniques du Languedoc; Guehl J-M *et al* (1998) *Oecol* 116:316–330; Hao G-Y *et al* (2008) *Oecol* 155:405–415; He J-S *et al* (2006) *New Phyt* 170:835–848; Holscher D (2004) *Bas Appl Ecol* 5:163–172; Huang J *et al* (2007) *For Ecol Man* 239:150–158; Karst AL & Lechowicz MJ (2007) *New Phyt* 173:306–312; Kenzo T *et al* 2004) *Tree Phys* 24:1187–1192; Kikuzawa K (1983) *Can J Bot* 61:2133–2139; Kleyer M *et al* (2008) *J Ecol* 96:1266–1274; Knapp A & Carter G (1998) *Am J Bot* 85:940–946; Kudo G *et al* (1999) *Ecoscience* 6:439–450; Li YL *et al* (2005) *Bot Bull of Acad Sini* 46:127–134; Margaris NS *et al* (1984) *Vegetatio* 55:29–35; McDonald PG *et al* (2003) *Func Ecol* 17:50–57; Milla R & Reich PB (2007) *Proc Biol Sci* 274:2109–2114; Miyazawa S-I *et al* (1998) *Ann Bot* 82:859–869; Mokany K & Ash J (2008) *J Veg Sci* 19:119–126; Navas ML *et al* (2003) *New Phyt* 159:213–228; Paula S & Pausas JG (2006) *Func Ecol* 20:941–947; Pierce S *et al* (2007) *J Ecol* 95:698–706; Prior LD *et al* (2003)

[1]Wright IJ *et al.* (2004) *Nature* 428: 821–827
[2]Kleyer *et al.* (2008) *J Ecol* 96: 1266–1274.

Func Ecol 17:504–515; Roche P *et al* (2004) *Plant Ecol* 174:37–48; Roderick ML *et al* (1999) *Func Ecol* 14:527–528; Roderick ML & Cochrane MJ (2002) *Ann Bot* 89:537–542; Roggy JC *et al* (1999) *Oecol* 120:171–182; Roumet C *et al* (1999) *New Phyt* 143:73–81; Royer DL *et al* (2007) *Paleobio* 33:574–589; Saldana-Acosta A *et al* (2008) *Act Oec* 34:111–121; Santiago L & Wright S (2007) *Func Ecol* 21:19–26; Saura-Mas S & Lloret F (2007) *Ann Bot* 99:545–554; Selaya NG & Anten N PR (2008) *Func Ecol* 22:30–39; Shipley B & Vu TT (2002) *New Phyt* 153:359–364; Tanner E VJ & Kapos V (1982) *Biotrop* 14:16–24; Taylor D & Eamus D (2008) 28:1169–1177; Thompson K *et al* (1997) *New Phyt* 136:679–689; Vaieretti MV, Díaz S, Vile D & Garnier E (2007) *Ann Bot* 99:955–958; Velazquez-Rosas N *et al* (2002) *Biotrop* 34:534–546; Vendramini F *et al* (2002) *New Phyt* 154:147–157; Vile D (2001) Master's thesis Université de Montpellier II; Vile D *et al* (2005) *Ann Bot* 96:1129–1136; Wang GH (2007) *J Veg Sci* 18:563–570; White JD & Scott NA (2006) *For Ecol Man* 226:319–329; Whitehead D & Walcroft AS (2005) *Ann For Sci* 62:525–535; Willert DJ *et al* (1990) *Plant Ecol* 90:133–143; Wilson PJ *et al* (1999) *New Phyt* 143:155–162; Wohlfahrt G *et al* (1999) *Plant Cell & Environment* 22:1281–1296; Würth M KR *et al* (2005) *Oecol* 143:11–24; Yang D *et al* (2008) *Ann Bot* 102:623–629; Zheng S & Shangguan Z (2007) *Plant Ecol* 191:279–293

Taxonomy and growth form:

Articles: Kluza-Wieloch *et al* (2005) *Rocz AR Pozn CCCLXXIII, Bot-Stec* 9:149–170; Mark AF *et al* (2001) *Austral Ecology* 26:423–440; Molau U *et al* (2005) *Am J Bot* 92:422–431;

Databases: Gachet S *et al* (2005) *Biol Conserv* 14:1023–1034; Kleyer M *et al* (2008) *Journal of Ecology* 96:1266–1274;

On-line resources:

Julve P (1998 –), Baseflor[3], version: 2008; US Forest Service, Pacific Island Ecosystems at Risk (PIER[4]) accessed 2008; Fire Effects Information System, US Department of Agriculture, Forest Service, Rocky Mountain Research Station, Fire Sciences Laboratory[5]; USDA, NRCS (accessed 2008) The PLANTS Database, National Plant Data Center [6]; Western Australian Herbarium (1998 –), Florabase - the western australian flora [7];

Paper floras: Allan HH Moore LB, Edgar E, Healy AJ, Webb C, Sykes WR, Garnock-Jones PJ & Connor, HE (1961) *Flora of New Zealand*; Govt Pr; Bentham, G & Bentham, G (1864) *Flora Australiensis: A Description of the Plants of the Australian Territory*; Clokey IW (1951) *Flora of the Charleston Mountains, Clark County, Nevada*, University of California Press; Hooker JD (1875) *The Flora of British India*, L Reeve & Co Ltd; Kihara H (1952) *Fauna and Flora Research Society Kyoto University, Kyoto* , 138–140; Lundell CL & Moldenke HN (1961) *Flora of Texas*, Texas Research Foundation; McCarthy PM (1999) *Flora of Australia Volume 17b Proteaceae 3 Hakea to Dryandra ABRS*, CSIRO, Melbourne; Munz PA & Keck DD (1959) *A California flora*, University of California Press, Berkeley; Ohwi J (1965) *Washington, DC*; Sell P & Murrell G (2006) *Flora of Great Britain and Ireland*, Cambridge University Press; Standley PC (1928) *Flora of the Panama Canal Zone*, US

[3] http://pagesperso-orange.fr/philippe.julve/catminat.htm
[4] http://www.hear.org/pier/
[5] http://www.fs.fed.us/database/feis
[6] http://plants.usda.gov
[7] http://florabase.calm.wa.gov.au/

Govt Print Off; Standley PC (1937) *Flora of Costa Rica*, Field Museum of Natural History; Wagner WL, Herbst DR & Sohmer SH (1990) *Manual of the Flowering Plants of Hawaii*, University of Hawaii Press; Welsh SL (1974) *Provo:Brigham Young Universisty Press xvi* 724p; Wu Z, Raven PH & Garden MB (1994) *Flora of China*, Science Press;

Unpublished: BD Ile-de-France; Plantes de l'Hérault

Published phylogenies:

Adoxaceae–Caprifoliaceae: Bell CD & Donoghue MJ (2005) *Am J Bot* 92:284–296; Bell CD & Donoghue MJ (2005) *Org Div Evol* 5:147–159; Caputo P *et al* (2004) *Plant Syst Evol* 246:163–175;

Amaranthaceae: Kadereit G *et al* (2003) *Int J Plant Sci* 164:959–986; Kadereit G *et al* (2005) *Org Div Evol* 5:59–80; Sage RF *et al* (2007) *Am J Bot* 94:1992–2003;

Anacardiaceae: Aguilar-Ortigoza C *et al* (2004) *Brittonia* 56:169–184; Pell SK (2004) PhD thesis;

Annonaceae: Doyle JA *et al* (2004) *Int J Plant Sci* 165:S55–S67; Richardson JE *et al* (2004) *Philos Trans R Soc Lond B Biol Sci* 359:1495–1508;

Araliaceae: Downie SR *et al* (2000) *Am J Bot* 87:273–292; Wen J *et al* (2001) *Syst Bot* 26:144–167;

Arecaceae: Asmussen CB *et al* (2006) *Bot J Linn Soc* 151:15–38; Baker WJ *et al* (1999) *Plant Syst Evol* 219:111–126;

Asteraceae: Fernàndez IA *et al* (2001) *Mol Phylogenet Evol* 20:41–64; Funk VA (2005) *Biol Skr* 55:343–373; Garcia-Jacas N *et al* (2002) *Mol Phylogenet Evol* 22:51–64; Hidalgo O *et al* (2006) *Ann Bot* 97:705–714; Kim H-G *et al* (2003) *Plant Syst Evol* 239:171–185; Kim HG *et al* (2002) *Syst Bot* 27:598–609; Panero JL & Funk V (2008) *Mol Phylogenet Evol* 47:757–782; Schmidt GJ & Schilling EE (2000) *Am J Bot* 87:716–726; Watson L *et al* (2002) *BMC Evol Biol* 2:1–12; Watson LE *et al* (2000) *Mol Phylogenet Evol* 15:59–69; Xiaoping Z & Bremer K (1993) *Plant Syst Evol* 184:259–283;

Betulaceae: Chen Z-D, Manchester SR & Sun H-Y (1999) *Am J Bot* 86:1168–1181;

Brassicaceae: Al-Shehbaz I *et al* (2006) *Plant Syst Evol* 259:89–120; Bailey CD *et al* (2006) *Mol Biol Evol* 23:2142–2160; Beilstein MA *et al* (2006) *Am J Bot* 93:607–619; Hall JC *et al* (2002) *Am J Bot* 89:1826–1842; Koch M *et al* (1999) *Plant Syst Evol* 216:207–230;

Mitchell AD & Heenan PB (2000) *Syst Bot* 25:98–105; Mummenhoff K *et al* (2001) *Am J Bot* 88:2051–2063; Ronse De Craene LP (2005) *Am J Bot* 92:752–760; Warwick SI & Sauder CA (2005) *Can J Bot* 83:467–483; Yang Y-W *et al* (1999) *Mol Phylogenet Evol* 13:455–462; Zunk K *et al* (1999) *Can J Bot* 77:1504–1512;

Caryophyllaceae: Fior S *et al* (2006) *Am J Bot* 93:399–411; Smissen RD *et al* (2002) *Am J Bot* 89:1336–1341;

Cunoniaceae: Bradford JC & Barnes RW (2001) *Syst Bot* 26:354–385;

Cyperaceae: Muasya AM *et al* (1998) *Plant Syst Evol* 211:257–271; Muasya AM *et al* (2001) *Syst Bot* 26:342–353; Roalson EH *et al* (2001) *Syst Bot* 26:318–341; Simpson DA *et al* (2003) *Am J Bot* 90:1071–1086; Verboom GA (2006) *Mol Phylogenet Evol* 38:79–89; Zhang X *et al* (2004) *Mol Phylogenet and Evol* 31:647–657;

Ericaceae: Anderberg AA *et al* (2002) *Am J Bot* 89:677–687; Kron KA (1996) *Ann Bot* 77:293–304; Kron KA (2002) *Bot Rev* 68:335–423; Schonenberger J *et al* (2005) *Int J Plant Sci* 166:265–288;

Fabaceae: Ainouche A *et al* (2003) Advances in legume systematics, part 10, 239–252; Allan GJ *et al* (2004) *Mol Phylogenet Evol* 32:123–138; Allan GJ *et al* (2004) *Syst Bot* 29:609–626; Bruneau A *et al* (2001) *Syst Bot* 26:487–514; Chandler GT *et al* (2001) *Am J Bot* 88:1675–1687; Choi H-K *et al* (2006) *Mol Genet Genom* 276:56–70; Crisp MD & Cook LG (2003) *Syst Bot* 28:705–713; Crisp MD *et al* (2000) Advances in legume systematics part 9, 249–276; Degtjareva G *et al* (2006) *Can J Bot* 84:813–830; Doyle J *et al* (1997) *Am J Bot* 84:541; Doyle J *et al* (2000) Advances in legume systematics, part 9, 1–20; Fougère-Danezan M *et al* (2007) *Syst Bot* 32:748–761; Haston EM *et al* (2005) *Am J Bot* 92:1359–1371; Hu J-M *et al* (2000) *Am J Bot* 87:418–430; Hu J-M, Lavin M, Wojciechowski MF & Sanderson MJ (2002) *Syst Bot* 27:722–733; Kajita T, Ohashi H, Tateishi Y, Bailey CD & Doyle JJ (2001) *Syst Bot* 26:515–536; Käss E & Wink M (1996) *Bioch Syst Ecol* 24:365–378; Käss E & Wink M (1997) *Mol Phylogenet Evol* 8:65–88; Lewis (2005) *Legumes of the world*; Royal

Botanical Garden Kew; Luckow M, Miller JT, Murphy DJ & Livshultz T (2003) *Advances in legume systematics* 10:197–220; McMahon M (2005) *Brittonia* 57:397–411; McMahon M & Hufford L (2004) *Am J Bot* 91:1219–1230; Moteetee A & van Wyk B-E (2006) *South Afr J Bot* 72:604–608; Orthia L, Cook L & Crisp M (2005) *Aust Syst Bot* 18:41–47; Osaloo SK, Maassoumi AA & Murakami N (2003) *Plant Syst Evol* 242:1–32; Pardo C, Cubas P & Tahiri H (2004) *Plant Syst Evol* 244:93–119; Pennington RT *et al* (2001) *Syst Bot* 26:537–556; Percy DM & Cronk Q CB (2002) *Am J Bot* 89:854–864; Sprent JI (2007) *New Phyt* 174:11–25; Wang HC *et al* (2006) *Bot J Linn Soc* 151:365–373; Wink M (2003) *Phytochemistry* 64:3–19; Wojciechowski MF *et al* (2004) *Am J Bot* 91:1846–1862;

Lauraceae: Chanderbali AS *et al* (2001) *Ann Miss Bot Gard* 88:104–134; Rohwer JG (2000) *Syst Bot* 25:60–71;

Liliaceae: Leitch IJ *et al* (2007) *J Evol Biol* 20:2296–2308;

Malvaceae: Alverson WS *et al* (1999) *Am J Bot* 86:1474–1486; Baum DA *et al* (2004) *Am J Bot* 91:1863–1871; Nyffeler R & Baum DA (2000) *Plant Syst Evol* 224:55–82; Nyffeler R *et al* (2005) *Org Divers Evol* 5:109–123; Tate JA *et al* (2005) *Am J Bot* 92:584–602; Whitlock BA (2001) *Syst Bot* 26:420–437; Wilkie P *et al* (2006) *Syst Bot* 31:160–170;

Melastomataceae: Clausing G & Renner SS (2001) *Am J Bot* 88:486–498; Fritsch PW, Almeda F, Renner SS, Martins AB & Cruz BC (2004) *Am J Bot* 91:1105–1114; Renner SS (1993) *Nord J Bot* 13:519–540; Stone RD (2006) *Syst Bot* 31:107–121;

Meliaceae: Muellner AN *et al* (2003) *Am J Bot* 90:471–480;

Moraceae: Richardson JE *et al* (2000) *Am J Bot* 87:1309–1324; Sytsma KJ *et al* (2002) *Am J Bot* 89:1531–1546; Zerega N JC *et al* (2005) *Mol Phylogenet Evol* 37:402–416;

Myrsinaceae-Primulaceae: Anderberg AA *et al* (1998) *Plant Syst Evol* 211:93–102; Hao G *et al* (2004) *Mol Phylogenet Evol* 31:323–339; Kallersjö M *et al* (2000) *Am J Bot* 87:1325–1341; Martins L *et al* (2003) *Plant Syst Evol* 237:75–85; Mast AR *et al* (2001) *Int J Plant Sci* 162:1381–1400;

Myrtaceae: Biffin M *et al* (2007) *Mol Phylogenet Evol* 43:124–139; Ladiges P *et al* (1999) *Cladistics* 15:151–172; Lucas EJ *et al* (2005) *Plant Syst Evol* 251:35–51; Sytsma KJ *et al* (2004) *Int J Plant Sci* 165:S85–S105; Wilson PG *et al* (2001) *Am J Bot* 88:2013–2025; Wilson PG *et al* (2005) *Plant Syst Evol* 251:3–19;

Oleaceae: Wallander E & Albert VA (2000) *Am J Bot* 87:1827–1841;

Onagraceae: Levin RA *et al* (2003) *Am J Bot* 90:107–115;

Orobanchaceae: Bennett JR & Mathews S (2006) *Am J Bot* 93:1039–1051; Park J-M *et al* (2008) *J Plant Res* 121:365–376; Tank DC & Olmstead RG (2008) *Am J Bot* 95:608–625;

Plantaginaceae–Scrophulariaceae: Albach DC & Chase MW (2001) *J Plant Res* 114:9–18; Albach DC *et al* (2005) *Am J Bot* 92:297–315; Beardsley PM & Olmstead RG (2002) *Am J Bot* 89:1093–1102; Estes D & Small RL (2008) *Syst Bot* 33:176–182; Ghebrehiwet M *et al* (2000) *Plant Syst Evol* 220:223–239; Hoggard RK *et al* (2003) *Am J Bot* 90:429–435; Munoz-Centeno LM, Albach DC, Sanchez-Agudo JA & Martinez-Ortega MM (2006) *Ann Bot* 98:335–350; Oxelman B *et al* (2005) *Taxon* 54:411–425; Rahmanzadeh R *et al* (2005) *Plant Biology* 7:67–78; Ronsted N *et al* (2002) *Bot J Linn Soc* 139:323–338; Vargas P *et al* (2004) *Plant Syst Evol* 249:151–172; Wagstaft SJ & Garnock-Jones PJ (1998) *New Zeal J of Bot* 36:425–438;

Poaceae: Barker N *et al* (2001) *Ann Miss Bot Gard* 88:373–457; Catalàn P *et al* (1997) *Mol Phylogenet Evol* 8:150–166; Catalàn P *et al* (2004) *Mol Phylogenet Evol* 31:517–541; Davis JI & Soreng RJ (2007) *Aliso* 23:335–348; Ge S *et al* (2002) *Am J Bot* 89:1967–1972; Gerhold P *et al* (2008) *J Ecol* 96:709–712; Giussani LM *et al* (2001) *Am J Bot* 88:1993–2012; Guo Y-L & Ge S (2005) *Am J Bot* 92:1548–1558; Kellogg EA (2001) *Plant Physiol* 125:1198–1205; King GJ & Ingrouille MJ (1987) *New Phyt* 107:633–644; Mathews S & Sharrock R (1996) *Mol Biol Evol* 13:1141–1150; Mathews S *et al* (2000) *Am J Bot* 87:96–107; Quintanar A *et al* (2007) *Am J Bot* 94:1554–1569; Soreng RJ *et al* (2007) *Kew Bulletin* 62:425–454; Watanabe M *et al* (1994) *J Plant Res* 107:253–261; Zhang W (2000) *Mol Phylogenet Evol* 15:135–146;

Polygonaceae: Frye A SL & Kron KA (2003) *Syst Bot* 28:326–332; Kim S-T & Donoghue MJ (2008) *Syst Bot* 33:77–86; Pennington RT *et al* (2004) *Philos Trans R Soc Lond B Biol Sci Soc B* 359:515–538;

Proteaceae: Hoot SB & Douglas AW (1998) *Aus Sys Bot* 11:301–320; Jordan GJ *et al* (2005) *Am J Bot* 92:789–796;

Ranunculaceae: Hoot SB (1995) *Plant Syst Evol (suppl)* 9:241–251; Johansson JT (1995) *Plant Syst Evol* 9:253–261; Johansson JT & Jansen RK (1993) *Plant Syst Evol* 187:29–49; Ro KE *et al* (1997) *Mol Phylogenet Evol* 8:117–127;

Rhamnaceae: Richardson JE *et al* (2004) *Philos Trans R Soc Lond B Biol Sci Society B: Biological Sciences* 359:1495–1508; Richardson JE *et al* (2000) *Am J Bot* 87:1309–1324; Sytsma KJ *et al* (2002) *Am J Bot* 89:1531–1546;

Rosaceae: Potter D (2007) *Plant Syst Evol* 266:5–43;

Sapindaceae: Harrington MG *et al* (2005) *Syst Bot* 30:366–382;

Solanaceae: Wink M (2003) *Phytochemistry* 64:3–19
