# Supplementary material for: An evolutionary perspective on leaf economics: phylogenetics of leaf mass per area in vascular plants
Source: Ecol Evol. 2014 Jul 1;4(14):2799–811. doi: 10.1002/ece3.1087 (PMC4130440; doi:10.1002/ece3.1087)
Supplement: Supplementary file 2 [file ece30004-2799-SD2.pdf]

Electronic Supplementary Material

Data. We collected data on LMA of vascular plants (*Tracheophyta*), and its inverse specific leaf area (SLA) which we converted to LMA, from 180 published and unpublished studies and electronic databases (see supporting information (SI) for the list of references added to the Glopnet (Wright et al., 2004) and LEDA (Kleyer et al., 2008) databases). We retained only data collected in the field for outer-canopy leaves measured following standardized protocols (Cornelissen et al., 2003), and for adult plants to limit ontogenic effects. Species-mean values were calculated for species with multiple records. Infra-species levels (subspecies, varieties or forms) were not distinguished. The sample set of species and sites represented a wide range of plant communities in most of the climates where vascular plants occur, from Arctic tundra to tropical forest, from hot to cold deserts, and from grassland to woodland. Special attention was devoted to taxonomic description and consistency. We delimited major clades of *Tracheophyta* according to a phylogenetic nomenclature (Cantino et al., 2007). Family names in *Angiospermae* were matched to the latest phylogeny by the Angiosperm Phylogeny Group, APGIII (Angiosperm Phylogeny Group III, 2009). For *Gymnospermae* and *Lycopodiophyta* (lycophytes), we referred to the Angiosperm Phylogeny website, APWeb (Stevens, 2001 onwards). For *Monilophyta* (ferns), we used the taxonomy of Smith et al. (2006). Family and genus names were checked for synonymy against the lists of the Kew Botanical garden, APGIII and APWeb. Binomial names were checked using the International Plant Names Index (IPNI, 2008).

In total, we obtained LMA values for 5401 species in 69 orders, 241 families and 1835 genera (see Fig. 2 for an overview of sample completeness). The resulting dataset contains 5239 species in *Angiospermae*, 81 in *Gymnospermae*, 74 in *Monilophyta* and 7 in *Lycopodiophyta*. Among sampled genera, 814 (44%) comprised at least two species. We checked that the variability in LMA occurred mostly between species compared to within species (across

sites): σ^2_{among} =16286 *vs* σ^2_{within} =1912. In order to limit the influence of extreme values, we performed comparative analyses on log-transformed LMA values that we hereafter note l.

Information on growth forms and Raunkiaer life forms (Raunkiaer, 1934) was collected from original studies, databases, and electronic and paper flora (see supporting information for references). The main growth forms were graminoid, forb and shrub–tree. We distinguished epiphyte, climber, succulent and aquatic (which corresponded to Raunkiaer's helophyte and hydrophyte life forms) growth forms. Ferns and lycophytes (*Selaginellaceae* and *Lycopodiaceae*) formed a distinct group. Leaf habit (deciduous/evergreen) and life-history (annual/perennial) were available for a subset of the species only.

The supertree. Phylogenetic relationships between species were described as an informal supertree at species level based on published phylogenies (Bininda-Emonds, 2004). We used the tree of Chaw et al. (2000) as a skeleton for relationships between the *Lycopodiophyta*, *Monilophyta*, *Gymnospermae* and *Angiospermae* clades, and between families within *Gymnospermae*. We then branched family-level trees: for the *Angiospermae*, we used the resolved family-level tree of Davies et al. (2004) corrected for synonymy according to the phylogeny of the Angiosperm Phylogeny Group Angiosperm Phylogeny Group III, 2009. The absent family *Rhipogonaceae* was added as a sister clade to the *Philesiaceae*, and *Platanaceae* were distinguished from *Proteaceae*. For *Monilophyta* and *Lycopodiophyta*, we used the trees from Smith et al. (2006) and Wikström and Kenrick (2001). Within 34 *Angiospermae* families, we resolved relationships between genera using published family phylogenies (see supporting information for references). In the two large *Asteraceae* and *Poaceae* families, we used tribe-level trees to branch genera within tribes as polytomies (nodes with more than two descending daughter clades). Conflicting positions of genera across references were resolved by setting a polytomy at higher level. Genera in the unresolved families were branched as polytomies on the family node. Species were branched as polytomies at genus level in all families.

The resulting supertree had 1675 ancestral nodes among which 58% were resolved as bifurcations (nodes with two daughter clades). Below the genus level, the percentage of bifurcations among ancestral nodes rose to 75%.

Ages of ancestral nodes and phylogenetic distance. In order to estimate branch lengths, we dated the ancestral nodes of the supertree in a two-step procedure. We first dated well-identified nodes in the supertree using published ages. A total of 187 (11%) ancestral nodes matched dated nodes from the literature. For clades in *Angiospermae*, ages were taken from a comprehensive update of divergence times based on the analysis of sequence data (Bell, Soltis, and Soltis, 2010). These estimates were obtained using a relaxed method allowing for heterogeneity in substitution rates along the phylogeny and a number of fossil ages as calibration points. We completed these ages with estimates derived from several sources for non-angiosperm clades (Anderson, Bremer, and Friis, 2005; Bremer, Friis, and Bremer, 2004; Janssen and Bremer, 2004; Wikström, Savolainen, and Chase, 2001). Age consistency was checked across references. In the second step, the tips of the supertree were assigned an age of 0. Remaining unknown ages were calculated as a combination of the two closest known ages, and branch length as the difference between ages at the two ends. The second step was achieved using the *bladj* module of the Phylocom software (Webb, Ackerly, and Kembel, 2007). Ages ranged from 535 Myr for the root of the tree, that is the basis of the *Tracheophyta* clade, to 2.2 Myr for three genera in the *Juglandaceae* family (*Carya*, *Juglans* and *Petrocarya*). Node age dates the oldest evolutionary split within the corresponding clade. We note $\mathbf{D}$ the phylogenetic distance matrix across all species, with diagonal elements $d_{ii} = 0$ and d_{ij} being the estimated age of the most recent common ancestor (MRCA) of species i and j (age of divergence between the lineages i and j).

Models of continuous trait evolution. We present here the models used in the analyses of LMA evolution. These models estimate parameters that describe macro-evolutionary patterns expected from micro-evolutionary processes (Hansen and Martins, 1996). First,

we considered the Brownian motion (BM) model, which assumes that the trait evolves by means of genetic drift independently along the branches of the tree and at constant rate σ independently (Freckleton, Harvey, and Pagel, 2002; Hansen and Martins, 1996): during an infinitesimal period dt, the variation in the trait value l is $dl = \sigma^2 dt$, where the parameter σ measures the magnitude of perturbations or drift (Hansen, 1997). In addition to drift, directional selection produces similar macro-evolutionary patterns when it occurs under environmental conditions fluctuating randomly and rapidly compared to evolutionary time (Diniz-Filho, 2001; Hansen and Martins, 1996). In the BM model, the expected variance in the trait value at a given ancestral node k linearly increases with the distance (period) from the root to the node, d_{rk}: $V_k = \sigma^2(d_{rk})$ (Diniz-Filho, 2001; Freckleton, Harvey, and Pagel, 2002). V_k also measures the expected covariance between the trait values of any two species (i, j) in clade k: $V_k = cov(l_i, l_j)$ where l_i is the trait value of species i: the older the divergence between those two species, the higher the covariance.

Second, we considered models of LMA evolution by stabilizing selection (Hansen, 1997). These models conform to evolution following an Orstein-Ulhenbeck (OU) process: the variation of the trait value during an infinitesimal period dt sums as the effects of drift, $\sigma^2 dt$ as in the BM model, and selection towards a phenotypic optimum θ: $dl = -\alpha(l - \theta) + \sigma^2 dt$. The parameter σ has the same interpretation as in the BM model. The parameter α controls the rate of adaptive evolution to the optimum θ, or the rate at which the past is discounted in the model: a descendant starts out being similar to its ancestor then the similarity exponentially decreases at a rate determined by α (Hansen, 1997). α is analogous to the magnitude of a supposed selective force. If α is large, species tend to adapt rapidly and trait values are weakly scattered around the optima, depending on σ; if α is small, the equilibrium variance among species is large and species tend to drift apart. In model OU, the expected covariance between two species i and j is $cov(l_i, l_j) = \frac{\sigma^2}{2\alpha} e^{-\alpha d_{ij}} \left[1 - e^{-2\alpha(d_r - dij)}\right]$, which shows the exponential decrease. Note that a null value of α leads to the BM model, which thus appears as

a special case of OU models.

The OU model allows different selective optima to be specified along one phylogenetic tree (Butler and King, 2004; Hansen, 1997): changes in the selective optimum mimic changes in the selection regime in different parts of the phylogeny. After a change in the selecting regime, evolution unfolds independently along each lineage. Here we considered three OU models based on different evolutionary scenarii following the approach of Butler & King (2004) (Butler and King, 2004) (see Fig. 5). Model OU1 specifies one single phenotypic optimum for the whole tree; model OU3 specifies three different optima thanks to a supposed change in the selection that occurred at the divergence at the basis of *Spermatophyta* and maintained two different optima along the *Angiospermae* and *Gymnospermae* branches; lastly, model OU5 includes the optima in OU3 and two others along the *Monocotyledoneae* and *Eudicotyledoneae* branches. Phenotypic optima were specified so that after divergence, different selection regimes occurred along the daughter branches (Fig. 5), instead of supposing that one of them further evolved under the regime before the split as in (Butler and King, 2004).

Models were fitted on the complete set of species and on the two sets of herbaceous and woody species separately by pruning the phylogenetic tree accordingly (Fig. S6). We distinguished the major herbaceous (forbs and graminoids) and woody (shrubs and trees) growth forms only in order to ensure sufficient numbers in each group. When dealing with OU models, parameter estimation can raise numerical issues (Butler and King, 2004; Hansen, 1997). We followed Hansen's recommendation (Hansen, 1997) to first estimate the α parameter and then run the calibration procedure for the other parameters (θ, σ) keeping parameter α fixed to its estimate. We compared the models using the Bayesian Information Criterion (BIC) calculated as BIC $= -2Ln(\mathcal{L}) + df_P Ln(n)$ where $\mathcal{L}$ is the likelihood of the model, n is the number of observations and df_P is the phylogeny-corrected number of degrees of freedom (Paradis and Claude, 2002): $df_P = \frac{b_\Sigma}{\sum_i^n d_{ri}}$ with b_Σ is the overall sum of the tree branch lengths and d_{ri} is the distance from the tree root to the tip i.

We also considered the null model of phylogenetically independent evolution (PI) which assumes that all species evolve independently as if they were placed at equal distance on a star-phylogeny. This model is not properly an evolutionary model in the sense that it ignores phylogenetic relatedness across species. It serves as a null hypothesis to test the existence of phylogenetic structure in the trait data.

Tree-level analysis of phylogenetic signal. We calculated four statistics to quantify the magnitude and test the significance of the phylogenetic signal at the tree level: Pagel's lambda (Pagel, 1999), the K-statistic (Blomberg, Garland, and Ives, 2003), the variance in phylogenetic independent contrasts (PIC) (Blomberg, Garland, and Ives, 2003) and phylogenetic autocorrelation measured by Moran's I (Gittleman and Kot, 1990). These statistics provide information on the likelihood of the phylogenetic pattern in LMA with respect to the PI and BM null hypotheses. In Pagel's approach, the parameter λ multiplies the off-diagonal (covariance) terms in the matrix $\mathbf{V}$: a value of $\lambda = 0$ indicates evolution independent of phylogeny (PI), while a value of $\lambda = 1$ indicates that the phylogenetic pattern conforms to Brownian motion (BM) on the given phylogeny (Freckleton, Harvey, and Pagel, 2002). We estimated λ by fitting a BM model using a maximum likelihood approach (Freckleton, Harvey, and Pagel, 2002).

In Blomberg *et al.*'s approach (Blomberg, Garland, and Ives, 2003), the K-statistic measures the degree to which a phylogenetic tree correctly describes the data, in fact the covariance structure observed in the data, compared to the null model of phylogenetic independence (PI): $K = \frac{r_{obs}}{r_{BM}}$ where r_{obs} is the ratio of the mean standard error (MSE) measured under PI to MSE derived from the covariance matrix $\mathbf{V}$ that describes the tree (Blomberg, Garland, and Ives, 2003):

$$r_{obs} = \frac{MSE_0}{MSE} = \frac{(\mathbf{X} - \hat{a})'(\mathbf{X} - \hat{a})}{(\mathbf{PX} - \hat{a})'(\mathbf{PX} - \hat{a})}$$

with $\hat{a}$ the estimated trait value at the root of the tree (Garland and Diaz-Uriarte, 1999) and

$\mathbf{P}$ satisfying the equation $\mathbf{PVP'} = \mathbf{I}$. A relatively high value of r_{obs} indicates that the phylogenetic tree correctly describes the covariance structure of trait values among the tips. In K's definition, r_{obs} value is divided by the value obtained under the hypothesis of Brownian motion evolution, r_{BM}, in order to allow comparison between studies (Blomberg, Garland, and Ives, 2003). The value of K was tested against the null hypothesis of phylogenetic independence (PI) by permuting the values among the tips a large number of times (Blomberg, Garland, and Ives, 2003). A K less than one indicates lower similarity among close relatives than expected under Brownian motion evolution along the candidate tree.

Finally, we perform an autocorrelation analysis at the tree level using Moran's index, I, which allows to quantify and test similarity in LMA values with respect to the phylogenetic distance between species (Diniz-Filho, 2001; Gittleman and Kot, 1990). Moran's I was calculated as:

$$I = \frac{n}{\sum_{i,j} \omega_{ij}} \frac{\sum_{i,j} \omega_{ij}(l_i - \bar{l})(l_j - \bar{l})}{\sum_i (l_i - \bar{l})^2}$$

where the sums are on the n species of the sample, l_i is the log-transformed LMA of species i, $\bar{l}$ is the mean trait value, and the weight ω_{ij} measures the degree of relatedness between species i and j. Weights were proportional to the inverse of the phylogenetic distance d_{ij} between species i and j, namely the age of their MRCA: $\omega_{ij} = \frac{1}{2d_{ij}}$. The value of I was also tested against the null hypothesis of phylogenetic independence (PI).

We also performed a correlogram analysis calculating I within classes of divergence time chosen to ensure a sufficient number of observations within each class. Phylogenetic correlograms indicate how the similarity among species, *i.e.* interspecific correlation, changes with the period of divergence between them. This temporal similarity pattern theoretically discriminates the BM and OU models (Diniz-Filho, 2001; Hansen and Martins, 1996): similarity is expected to decrease linearly with time since divergence when the trait evolves by random genetic drift only or by drift and rapidly changing directional selection (BM),

whereas it decreases exponentially when the trait evolves by drift and stabilizing selection (OU) (Hansen and Martins, 1996). We adjusted linear and exponential fits to the observed patterns in LMA similarity using non-linear least squares and compared the fits using a likelihood ratio test. Similar analyses were performed within growth forms. Phylogenetic correlograms were also calculated using taxonomic levels, which showed consistent patterns compared to the divergence time approach (see Fig. S5).

Clade-level analysis of the phylogenetic signal. We analyzed the phylogenetic signal at clade level using an Analysis Of Traits (AOT Webb, Ackerly, and Kembel, 2007). This analysis can detect ancestral divergences in the phylogenetic tree which correspond to conservative or diversifying splits in LMA values across daughter clades (Ackerly and Nyffeler, 2004; Moles et al., 2005) (see supplementary data and results of AOT in SI). In AOT, the divergence width statistic, DW, measures the divergence size (or trait radiation) in the ancestral mean, a, which is an estimated value of the trait for each ancestral node. DW is calculated as the standard deviation of a across daughter clades with respect to the ancestor's value of a:

$$DW_i = \left(\frac{1}{n_i} \sum_{j=1}^{n_i} (a_{i,j} - a_i)^2 \right)^{\frac{1}{2}}$$

where n_i is the number of daughter nodes of ancestral node i, a_i its estimated mean and $a_{i,j}$ the estimated mean of daughter node j. The ancestral mean (a) was estimated recursively from the tips' attributes to the root of the tree following Felsenstein's algorithm (Felsenstein, 1985):

$$a_i = \frac{\sum_{j=1}^{n_i} \frac{a_{i,j}}{b_{i,j}}}{\sum_{j=1}^{n_i} \frac{1}{b_{i,j}}}$$

where $b_{i,j}$ is the branch length between nodes i and j. Significant divergence widths were detected by testing the statistic against the null hypothesis PI. As for tree-level statistics, the test was conducted by permuting LMA values among the tips (n = 100000 permutations). P-values were corrected using the Benjamini & Hochberg method (Benjamini and Hochberg,

1995) for multiple testing, ensuring a stringent analysis. When the DW of a node was lower than expected at random, the node was characterized as conservative; when the DW value was higher than expected at random, the node was characterized as diversifying.

Supporting figures

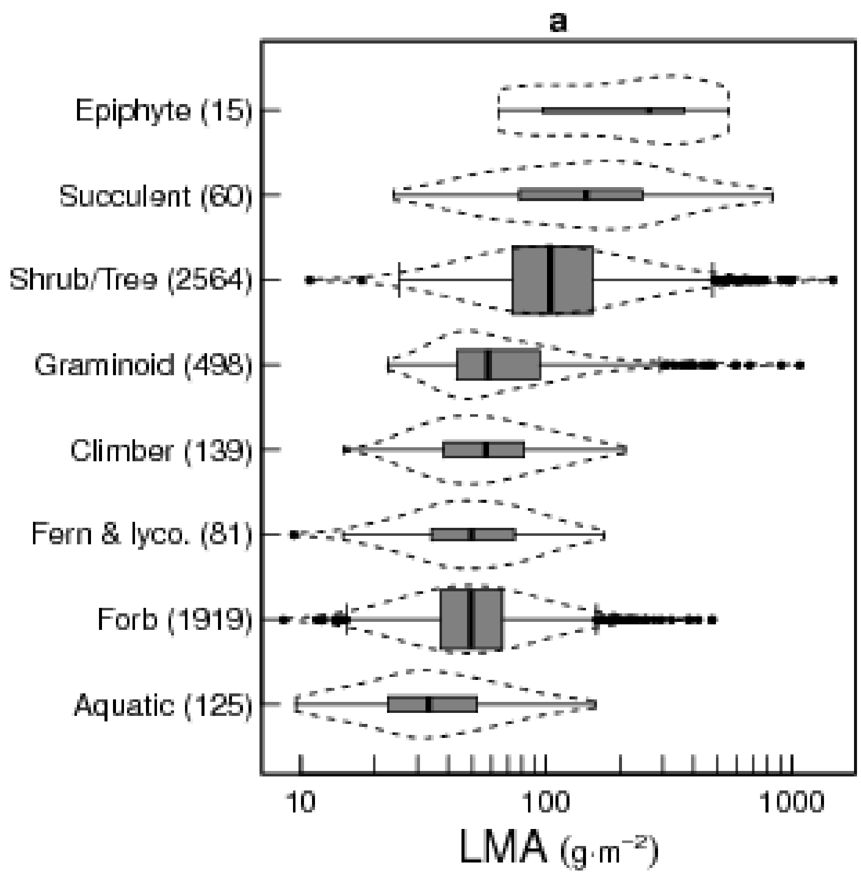

Fig. S1. Leaf Mass per Area (LMA) within growth forms. Distribution of LMA (g.m^{-2}) with respect to the different growth forms in order of increasing mean LMA values. In brackets: n, number of species per growth form (Table S1). Boxes and vertical lines indicate the interquartile range and the median in each category. Box height is proportional to $\sqrt{n}$. Dotted lines represent the smoothed distribution within each category, and crosses indicate outliers.

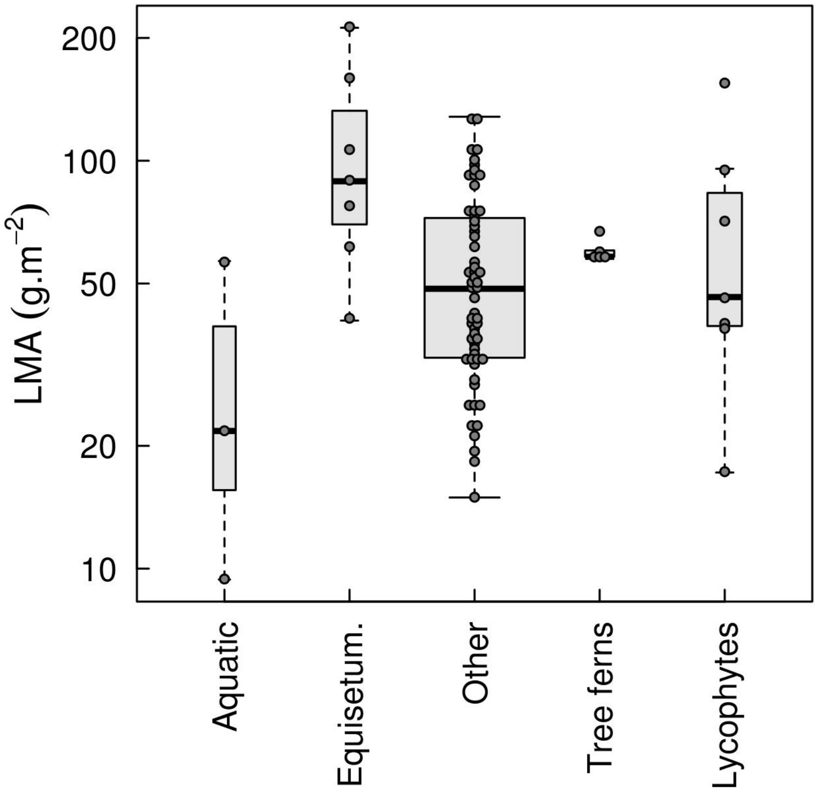

Fig. S2. Detailed distribution of Leaf Mass per Area (LMA) within ferns and lycohytes.

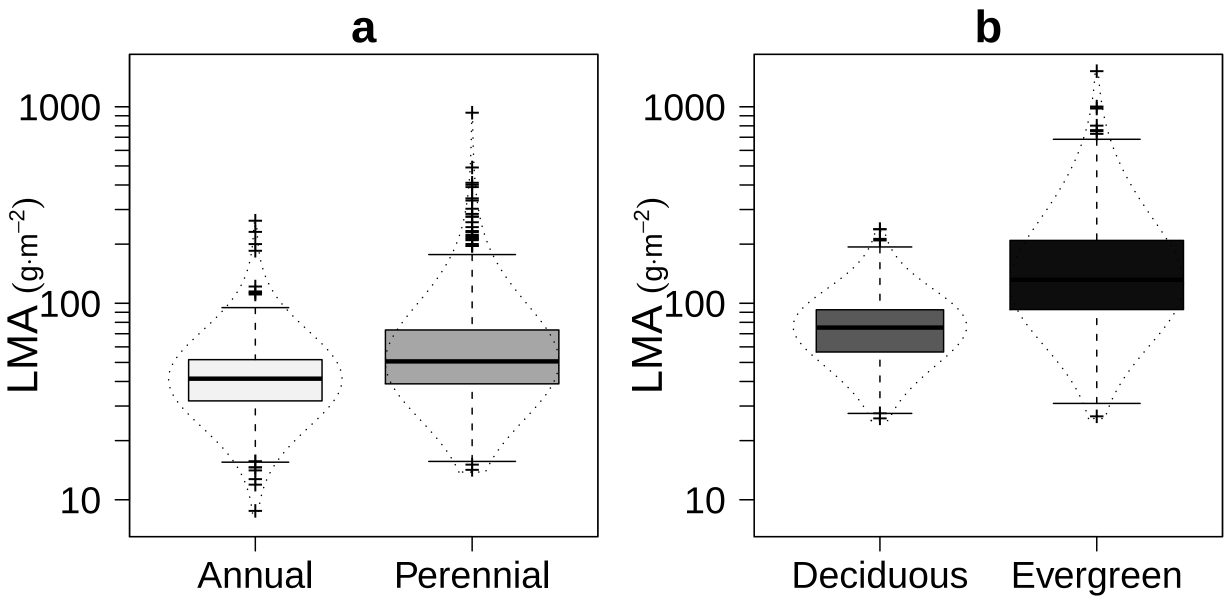

Fig. S3. Leaf Mass per Area (LMA) in the subsample of (a) herbaceous species for which life history was unambiguous (annual: mean: 45g.m^{-2}/ coefficient of variation: /0.51, $n =$ 545; and perennial : 65/0.82, $n = 921$) and of (b) woody species for which leaf phenology was unambiguous (deciduous : 79/0.38, $n = 522$; evergreen : 174/0.76, $n = 969$). Dotted lines indicate the shape of the distribution within each category. Box width is proportional to the number of species in the corresponding category.

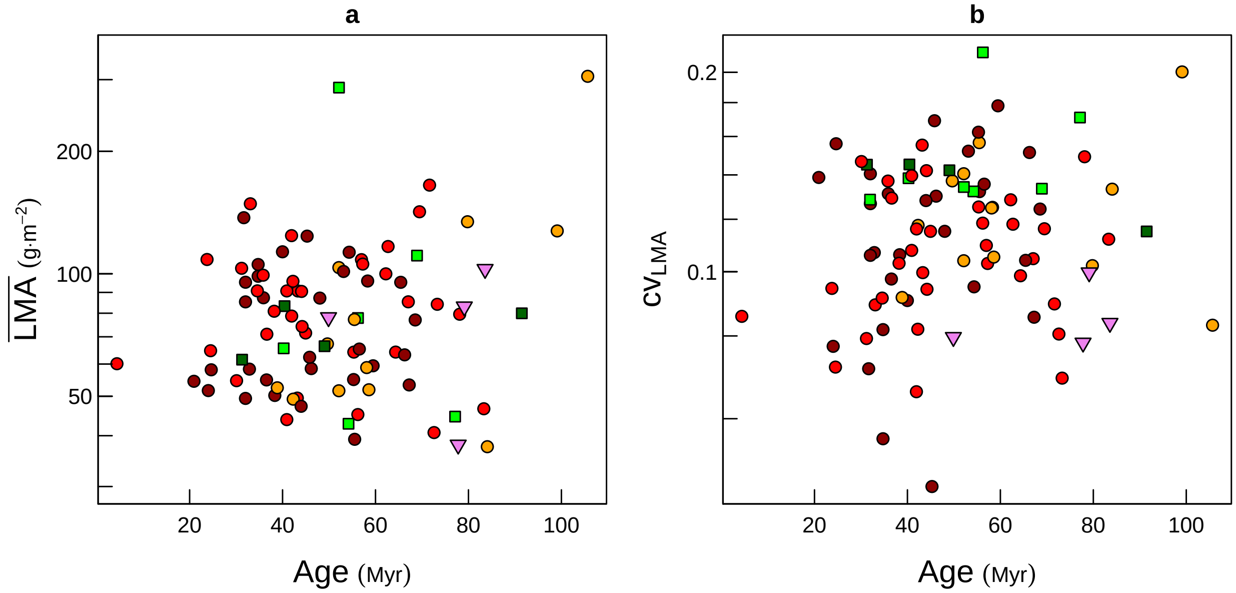

Fig. S4. LMA at the level of families: (a) Mean LMA ($\overline{\text{LMA}}$ in g.m^{-2}) across extant species within families *vs* estimated age of families (in Myr); (b) Coefficient of variation of LMA within families *vs* estimated age. Symbols and colors indicate major clades of angiosperms: ○ *Eudicotyledoneae* (orange: basal families, red: *Asteridae*, dark red: *Rosidae*); □ *Monocotyledoneae* (green: basal families; dark green: *Commelinidae*); ▽ *Magnoliidae*. Only families with over 10 sampled species were considered.

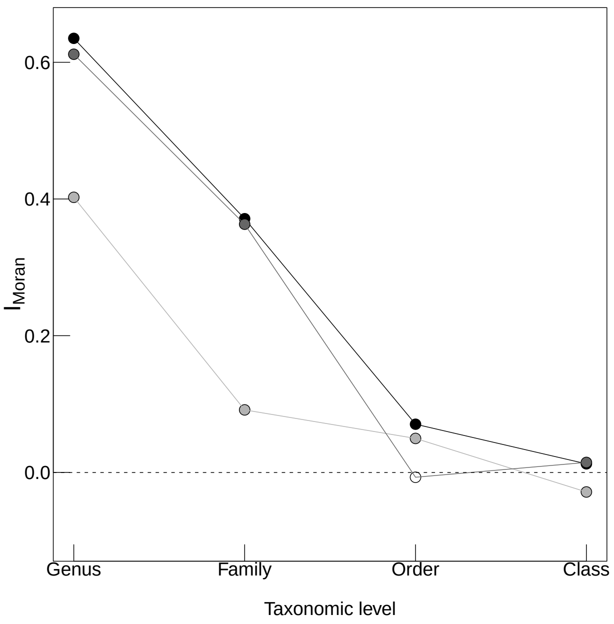

Fig. S5. Correlogram of LMA showing Moran's I (I_M) within taxonomic levels, for the complete sample (black line and symbols, n =5401 species), and for the woody (dark gray, n =2564 species) and herbaceous (light gray, n =2417 species) growth forms. Filled symbols indicate significant autocorrelation, open symbols non-significant autocorrelation.

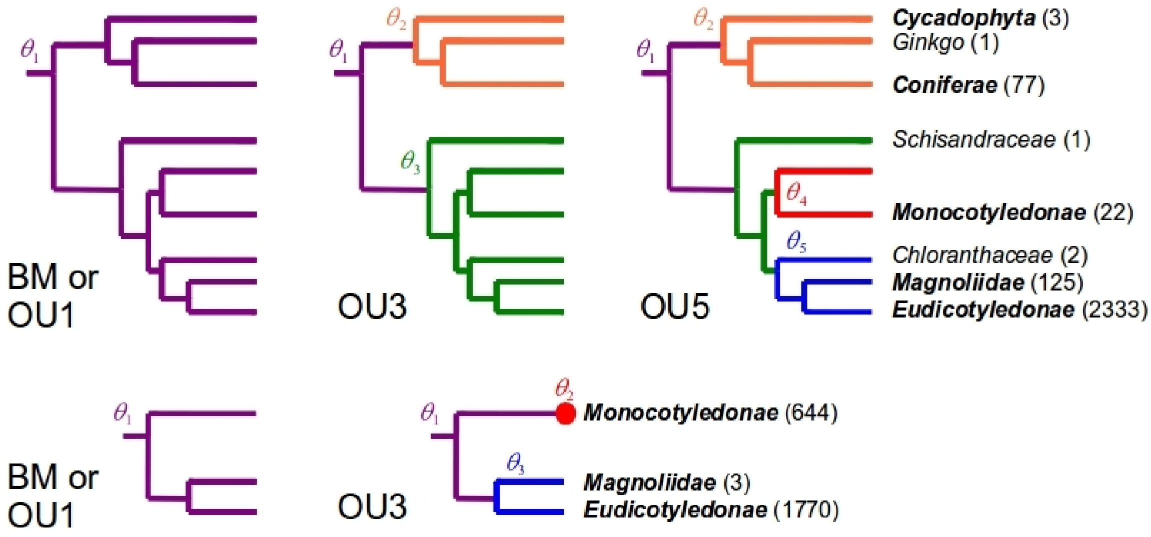

Fig. S6. Alternative models for LMA evolution in woody (top) and herbaceous species (bottom). BM : Brownian motion; OU : Ornstein-Uhlenbeck with one (1), three (3), or five (5) optima. Colors indicate different phenotypic optima in LMA within clades. Only major and particular basal clades and their sister clades are represented (arbitrary branch lengths; clade size in parentheses).
