# Supplementary material for: An evolutionary perspective on leaf economics: phylogenetics of leaf mass per area in vascular plants
Source: Ecol Evol. 2014 Jul 1;4(14):2799–811. doi: 10.1002/ece3.1087 (PMC4130440; doi:10.1002/ece3.1087)
Supplement: Supplementary file 3 [file ece30004-2799-SD3.pdf]

Table S1. Growth forms and clades. Occurrence table of sampled taxa within major Tracheophytes clades (Letter "b." indicates basal clades, *e.g. Chloranthaceae, Nymphaeaceae* and *Schisandraceae* in *Angiospermae*) and with respect to growth form. Fe/Ly: ferns and lycophytes, Aqu.: aquatic (Raunkiaer's helophytes and hydrophytes), Climb: climber, Epi: epiphyte, Succ: succulent, Gram: graminoid, Sh./Tr.: shrub, tree or intermediate. Notes: [1] *Ceratophyllum demersum*, the only species sampled in the *Ceratophyllaceae* family is included here in the basal *Monocotyledonae*, but placed as a sister clade to the *Monocotyledonae* in the phylogeny (see Fig. 2); [2] *Stylidium lineare* (*Stylidiaceae*). n_{total}: number of species sampled in each category; Mean LMA: mean LMA in g.m^{-2} in each category; C.V. LMA: coefficient of variation of LMA in each category;

	Fe/Ly	Aqu	Climb	Epi	Succ	Gram	Forb	Sh/Tr	n_{total}	Mean LMA (g.m^{-2})	C.V. LMA
Lycopodiohyta	7								7	66	0.7
Monilophyta (ferns)	74								74	59	0.6
Gymnospermae								81	81	238	0.58
b.*Angiospermae*		2						3	5	69	0.35
b.*Monocotyledoneae*[1]		42	8	1	4	18	132	6	210	90	1.48
Commelinidae		29	1	4		479	15	16	544	84	1
Magnoliidae			6				3	125	134	95	0.45
b.Eudicots		12	18	4	43		336	320	733	158	1.05
Rosidae		9	62	3	6		490	1389	1959	99	0.78
Asteridae		31	44	3	7	1[2]	943	624	1653	78	0.67
n_{total}	81	125	139	15	60	498	1919	2564	5401		
Mean LMA	59	43	66	244	198	91	58	137			
C.V. LMA	0.6	0.69	0.58	0.64	0.86	1.13	0.64	0.82			

Table S2. Results from family-level linear models of the mean and coefficient of variation (CV) of log-transformed mean LMA values, l, within *Eudicotyledonae* families. Relationships were estimated under two hypotheses of trait evolution using Generalized Least Squares (GLS): phylogenetic independence (PI) as in non-phylogenetic linear models, and Brownian motion (BM) which supposes evolution at constant rate along independent lineages. BM models were estimated using untransformed branch lengths or simultaneously estimating Pagel's parameter λ for branch length transformation (BM_λ). a) Model comparison: BIC: Bayesian Information Criterion, $ln(\mathcal{L})$: log-likelihood, σ: residual error, λ: Pagel's parameter (1 in the BM case). b) Estimated values of coefficients in the models, standard errors, t-statistics and p-values. Explicative variables were: estimated age of divergence (Age in Myr), proportion of woody species within the family (Woody in %), and clade size, (N, number of sampled species). Only families with ≥ 10 species were considered ($n = 91$).

a.

	BIC	$ln(\mathcal{L})$	σ	λ
**Mean** l				
PI	112.7	-45.2	0.359	
BM	**110.4**	-44.0	0.479	1
BM_λ	110.6	-41.9	0.411	0.750
**CV** l				
PI	**43.1**	-10.4	0.241	
BM	65.9	-21.776	0.371	1
BM_λ	47.5	-10.4	0.239	-0.047

b.

	Value	Std.Error	t	p
**Mean** l				
**PI**				
Int.	3.761	0.194	19.4	$< 10^{-4}$
Age (Myr)	0.004	0.002	1.8	0.079
Woody (%)	0.604	0.096	6.3	$< 10^{-4}$
log(N)	0.013	0.042	0.3	0.760
**BM**				
Int.	3.875	0.223	17.3	$< 10^{-4}$
Age (Myr)	0.003	0.002	1.4	0.171
Woody (%)	0.647	0.112	5.8	$< 10^{-4}$
log(N)	−0.032	0.039	−0.8	0.411
$\mathbf{BM_\lambda}$				
Int.	3.788	0.214	17.7	$< 10^{-4}$
Age (Myr)	0.003	0.002	1.6	0.113
Woody (%)	0.678	0.107	6.3	$< 10^{-4}$
log(N)	−0.014	0.039	−0.3	0.728
**CV** l				
**PI**				
Int.	−2.130	0.130	−16.4	0.000
Age (Myr)	0.002	0.001	1.4	0.164
Woody (%)	−0.335	0.064	−5.2	$< 10^{-4}$
log(N)	0.010	0.028	0.4	0.716
**BM**				
Int.	−2.209	0.173	−12.8	$< 10^{-4}$
Age (Myr)	0.003	0.002	1.6	0.109
Woody (%)	−0.272	0.087	−3.1	0.002
log(N)	−0.008	0.030	−0.3	0.786
$\mathbf{BM_\lambda}$				
Int.	−2.125	0.128	−16.6	$< 10^{-4}$
Age (Myr)	0.002	0.001	1.4	0.166
Woody (%)	−0.344	0.061	−5.6	$< 10^{-4}$
log(N)	0.010	0.028	0.4	0.709

Table S3. Summary of evolution models for LMA. Models were fitted on the complete species set (All) and separately within the two main growth form groups (Herbaceaous and Woody) using pruned trees (Fig. S6). n: number of considered species in each case, df_P: phylogeny-corrected number of degrees of freedom (see main text for details), μ phylogeny-corrected mean LMA in the Brownian motion model (BM). σ: residual error, also interpreted as the magnitude of stochastic noise resulting from drift. In models with stabilizing selection (Ornstein-Uhlenbeck models, OU), θ_i indicates different phenotypic optima occurring in different parts of the phylogeny, which correspond to different selective regimes (see Fig. 4 & S6 for optima specification). α: adaptation rate to phenotypic optima. $-\ln(\mathcal{L})$: negative log-likelihood. BIC: Bayesian Information Criterion.

	All	Herbaceous	Woody
n	5401	2417	2564
df_P	259	304	254
**Brownian motion (BM)**			
μ	61	50	128
σ	2.616	1.567	1.637
$-\ln(\mathcal{L})$	4334	1870	1884
BIC	10896	6112	5759
**Single optimum (OU1)**			
α	0.062	0.062	0.028
θ_1	81	54	112
σ	0.581	0.530	0.487
$-\ln(\mathcal{L})$	4355	3863	1419
BIC	10938	10098	4829
**Three optima (OU3)**			
α	0.062	0.045	0.028
θ_1	79	65	105
θ_2	201	49	178
θ_3	51	50	110
σ	0.568	0.508	0.483
$-\ln(\mathcal{L})$	4240	1425	1401
BIC	10707	**5221**	**4793**
**Five optima (OU5)**			
α	0.062		0.028
θ_1	82		104
θ_2	65		106
θ_3	62		58
θ_4	201		178
θ_5	51		109
σ	0.321		0.233
$-\ln(\mathcal{L})$	4222		1400
BIC	**10670**		**4791**

Table S4. Genera associated with significant conservative divergences. Numbers in parentheses indicate the number of sampled species in each genus.

Order	Family	Genus (♯ sampled species)
Asterales	Asteraceae	*Carlina* (4), *Centaurea* (19), *Cirsium* (15), *Crepis* (13), *Eupatorium* [illegible]
Asparagales	Orchidaceae	*Dactylorhiza* (8)
Brassicales	Brassicaceae	*Cochlearia* (5)
Caryophyllales	Caryophyllaceae	*Cerastium* (16), *Sagina* (6), *Silene* (20)
	Polygonaceae	*Rumex* (15)
Dipsacales	Caprifoliaceae	*Lonicera* (17)
Ericales	Ebenaceae	*Diospyros* (16)
	Ericaceae	*Pyrola* (4), *Rhododendron* (15)
	Lecythidaceae	*Gustavia* (2)
	Myrsinaceae	*Heberdenia* (2)
Fabales	Fabaceae	*Trifolium* (29), *Vicia* (15)
Fagales	Betulaceae	*Alnus* (13), *Betula* (17)
	Fagaceae	*Quercus* (60)
Gentianales	Geraniaceae	*Geranium* (17)
Lamiales	Orobanchaceae	*Pedicularis* (13), *Rhinanthus* (3)
	Scrophulariaceae	*Eremophila* (8)
	Euphorbiaceae	*Macaranga* (14)
	Salicaceae	*Populus* (16), *Salix* (55)
Myrtales	Myrtaceae	*Eucalyptus* (57)
Poales	Cyperaceae	*Carex* (107)
	Poaceae	*Bromus* (19), *Setaria* (6)
Polypodiales	Dryopteridaceae	*Dryopteris* (9)
Proteales	Proteaceae	*Banksia* (11), *Hakea* (99), *Protea* (16)
Ranunculales	Ranunculaceae	*Ranunculus* (36)
Rosales	Moraceae	*Ficus* (18)
	Rosaceae	Potentilla (43), *Rosa* (27), *Rubus* (18), *Sorbus* (14)
Sapindales	Sapindaceae	*Acer* (24)
